# Reinforcing the Efficiency of Plastic Upgrading through Full‐Spectrum Photothermal Effect Integration of Heat Isolator

**DOI:** 10.1002/advs.202410260

**Published:** 2024-10-28

**Authors:** Xueqin Gong, Peng Wang, Shuo Yang, Wenbo Li, Min Lv, Bei Li, Xiangxiang Zhang, Zeyan Wang, Yuanyuan Liu, Peng Wang, Hefeng Cheng, Ying Dai, Baibiao Huang, Zhaoke Zheng

**Affiliations:** ^1^ State Key Laboratory of Crystal Materials Shandong University Jinan 250100 China; ^2^ School of Chemistry and Chemical Engineering Shandong University Jinan 250100 China; ^3^ School of Physics Shandong University Jinan 250100 China

**Keywords:** alkali‐free hydrothermal pretreatment, heat isolator, photothermal reforming of PET

## Abstract

Photoreforming of polyethylene terephthalate (PET) to H_2_ is practically attractive strategy for upgrading waste plastics. The major challenge is to utilize the infrared energy in the solar spectrum to improve the efficiency for photoreforming of PET to H_2_. Herein, through the ingenious integration of tungsten phosphide nanoparticles and tungsten single atoms (WP/W SAs) with carbon nitride (g‐C_3_N_4_), the constructed hybrid inherits both the desirable properties and structural merits of the respective building blocks. Specifically, the photothermal effect of WP/W SAs couples with the “heat isolator” role of g‐C_3_N_4_ due to its low thermal conductivity, thereby forming localized high‐temperature regions, reducing the activation energy and improving the kinetics in the photoreforming of PET to H_2_. Additionally, the green pretreatment of PET using alkali‐free hydrothermal strategy is reported, achieving direct separation of the ethylene glycol and terephthalic acid. This work not only provides an alkali‐free hydrothermal pretreatment for PET, but also integrates the photothermal effect with the thermal insulation and opens a new avenue for harnessing solar energy into to convert plastics into H_2_.

## Graphical abstract

To enhance the activity for photoreforming of PET to H_2_, the photothermal effect of WP/W SAs couples with the “heat isolator” role of g‐C_3_N_4_ due to its low thermal conductivity, thereby forming localized high‐temperature regions and reducing the activation energy. Additionally, a strategy of alkali‐free hydrothermal pretreatment of PET is reported, achieving direct separation of ethylene glycol and terephthalic acid.

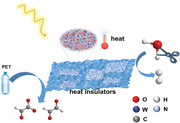

## 1 Introduction

Plastics are one of the most widely used materials in our lives due to their desirable properties, such as lightness, durability, and low price.^[^ ^1^, ^2^, ^3^, ^4^ ^]^ However, with the increase of white pollution, the disposal of plastics is not only a serious threat to human health, but also a drastic waste of carbon resources. Although conventional technologies such as landfill, incineration, and pyrolysis methods have been widely reported for reusing the discarded plastics,^[^ ^5^, ^6^ ^]^ it is necessary to develop novel and efficient strategies to degrade or recycle plastic waste under mild conditions. Recently, photocatalytic plastics upcycling has attracted considerable attention from the scientific community.^[^ ^7^, ^8^ ^]^ Especially, photoreforming of plastics into carbonaceous fuels and H_2_ fuels is considered to be one of the most promising strategies.^[^ ^4^, ^9^ ^]^ However, the solar energy in the infrared region is wasted because the photoelectric excitation of semiconductor materials alone is not sufficient to cover the entire spectrum of solar energy.^[^ ^10^, ^11^ ^]^ To overcome the above problems, the use of sunlight‐converted thermal energy to drive thermal catalysis, also known as solar thermal catalysis, can efficiently drive the photocatalytic hydrogen production from water.^[^ ^12^ ^]^ Photothermal catalysis typically combines the advantages of traditional thermal catalysis and photocatalysis, and the synergistic effect of these two catalytic processes improves performance.^[^ ^11^, ^13^ ^]^ Additionally, previous reports have shown that PET plastics require alkaline pretreatment before photo reforming, which not only causes environmental pollution but also makes it impossible to recover terephthalic acid derived from plastics in solution.^[^ ^14^, ^15^ ^]^ Thus, it is urgent to find an environmentally friendly and fully utilizing method for the pre‐treatment of PET plastics. The use of water without alkali as a solvent for high‐temperature hydrothermal treatment is an environmentally friendly method for the pretreatment of plastics. In addition, the polarity of water molecules was decreased in subcritical water due to the average sharing of electrons between oxygen and hydrogen atoms.^[^ ^16^ ^]^ The increased decomposition of water into H^+^ and OH^−^ ions can drive acid or base catalytic reactions. Therefore, PET plastics can be quantitatively depolymerized into ethylene glycol (EG) and terephthalic acid (PTA) in supercritical water in this work.

In recent years, transition metal phosphates (TMPs) have been developed as potential co‐catalysts for photocatalytic hydrogen evolution.^[^ ^17^ ^]^ WP nanoparticles (WP NPs) have received more attention in electrochemical HER,^[^ ^18^, ^19^, ^20^ ^]^ while WP NPs as co‐catalyst in photothermal reforming of plastic to H_2_ is far from satisfactory. Compared to nanoparticles, single‐atom catalysts are the most promising strategy in chemical conversions due to high atomic efficiency,^[^ ^21^ ^]^ tunable atomic configuration, and electronic metal‐support interaction. W single atoms, as a replacement for expensive platinum‐based catalysts, are attractive candidates for accelerating water splitting kinetics.^[^ ^20^, ^21^ ^]^ Therefore, the coupling of WP NPs and W single atoms (W SAs) can modulate the charge redistribution of WP through the interface between adjacent single sites and WP NPs, which can reduce the kinetic energy barrier in the photothermal reforming of plastics to H_2_. Additionally, TMPs exhibit photothermal properties due to their mobile valence electrons and large light absorption cross sections.^[^ ^17^, ^22^, ^23^ ^]^ Here, we consider the potential of WP/W SAs to promote the acceleration of charge transfer kinetics through photothermal effects. To the best of our knowledge, there have been no reports on exploiting the synergistic function of WP/W SAs to achieve photothermal reforming of PET. Although WP NPs and W SAs as photothermal materials can absorb almost the entire solar spectrum, the catalytic activity is low due to the rapid heat loss to the surrounding environment and the recombination of photogenerated carriers.^[^ ^24^ ^]^ Therefore, how to reduce the heat loss of materials is the key to improving photothermal reforming plastics. Previous studies indicated that the photocatalyst with ultralow thermal conductivity can greatly suppress the heat loss to the surroundings.^[^ ^25^, ^26^ ^]^ g‐C_3_N_4_ is among the most extensively studied semiconductor photocatalysts due to structural versatility, tunable electronic and optical properties, and chemical stability.^[^ ^27^, ^28^ ^]^ Although the theoretical thermal conductivity of g‐C_3_N_4_ is relatively high,^[^ ^29^ ^]^ the thermal conductivity of porous g‐C_3_N_4_ is close to that of air, suggesting that porous g‐C_3_N_4_ has great potential for suppressing heat loss.^[^ ^30^ ^]^ In addition, the ultra‐thin porous g‐C_3_N_4_ nanosheets as a photocatalyst can provide more active sites, shorter diffusion paths, and better mass transfer.^[^ ^31^, ^32^ ^]^

The synergistic interaction of W SAs and WP NPs can enhance the hydrogen evolution performance of g‐C_3_N_4_ in the photothermal reforming of PET. On the one hand, the photothermal effect induced by WP NPs can be rapidly heated to a temperature range where thermal catalytic reactions are possible. Meanwhile, the porous structure of g‐C_3_N_4_ can minimize the heat loss as much as possible. Therefore, the increase in the near‐field temperature around the g‐C_3_N_4_ is due to the local solar heat caused by the insulation of g‐C_3_N_4_, which accelerates the photochemical reaction and synergistically promotes the integrated conversion of reactions. On the other hand, W SAs can promote water activation, which was confirmed by single particle PL spectroscopy and DFT calculations. More importantly, the direct separation of EG andPTA in plastics has been achieved through the strategy of hydrothermal plastics, which fully utilizes the substances in plastics. The findings of this work will pave the way for the development of low‐cost and high‐efficiency catalysts for photothermal reforming of plastic waste.

## 2 Results and Discussion

### 2.1 Morphological and Physical Phase Structure Characterization

**Figure 1:**
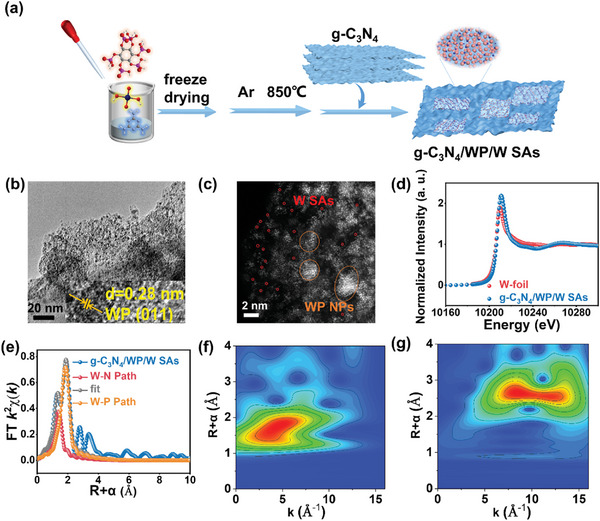
a) Schematic of the synthesis procedure of g‐C_3_N_4_/WP/W SAs and g‐C_3_N_4_/WP. b) High‐resolution TEM (HRTEM) and c) HAADF‐STEM image of g‐C_3_N_4_/WP/W SAs. d) XANES of g‐C_3_N_4_/WP/W SAs and W foil. e) The Fourier transform EXAFS spectrum of g‐C_3_N_4_/WP/W SAs. WT EXAFS of f) g‐C_3_N_4_/WP/W SAs and g) W foil.

As shown in **Figure** **1**a, we propose to use freeze‐drying to prepare the precursors. The freeze‐drying process can limit the dispersion of the reactants and prevent nucleation.^[^ ^33^, ^34^ ^]^ At this stage, instead of the conventional dispersion of W ions in bulk water, W ions were confined by the ice template localized on the substrate surface. After removing the ice template by vacuum freeze‐drying, the precursor of W ions uniformly adsorbed on the surface can be obtained,^[^ ^35^ ^]^ and finally W SAs can be grown at high temperature. WP/W SAs and g‐C_3_N_4_ were coupled by 2D/2D van der Waals forces to obtain g‐C_3_N_4_/WP/W SAs. For comparison, we synthesized WP NPs by direct drying method and prepared g‐C_3_N_4_/WP by the same procedure. The structure of WP/W SAs and WP NPs showed no obvious changes, suggesting that no chemical reaction involved during the freeze‐drying process (Figure S1, Supporting Information). Furthermore, Wang et al. used high‐angle annular dark‐field scanning transmission electron microscopy (HAADF‐STEM) to demonstrate the absence of W single atoms through direct drying methods.^[^ ^36^ ^]^ Obviously, the WP NPs in g‐C_3_N_4_/WP/W SAs were smaller in size and more homogeneously dispersed (Figure 1b; Figure S2, Supporting Information). These results suggested that the precursor generated by the freeze‐drying technology can form mesoporous structure during the thermal polymerization process, which may promote the uniform distribution of metal ions on the surface of melamine. The ultra‐small WP NPs were ≈5 nm in diameter with highly uniform dispersion, and the WP lattice fringes (with ad‐spacing of 0.28 nm) were assigned to the (011) lattice planes. To determine the distribution of the W SAs, HAADF‐STEM measurement was performed. Figure 1c showed the co‐existence of highly dispersed bright dots (W SAs) and nanoparticles (WP NPs) in g‐C_3_N_4_/WP/W SAs. Additionally, the HAADF‐STEM‐EDS image of g‐C_3_N_4_/WP/W SAs indicated a uniform elemental distribution of W, N, C, and P (Figure S3, Supporting Information).

To investigate the coordination configurations of g‐C_3_N_4_/WP/W SAs, X‐ray absorption near‐edge structure (XANES) and extended X‐ray absorption fine structure spectroscopy (EXAFS) were conducted. As shown in Figure 1c, the absorption edge energy of g‐C_3_N_4_/WP/W SAs showed obviously positive shift compared to that of W foil (Figure 1d), suggesting that the average oxidation state of W in the g‐C_3_N_4_/WP/W SAs was more positive due to the presence of nitrogen.^[^ ^20^ ^]^ The Fourier transform EXAFS spectrum of g‐C_3_N_4_/WP/W SAs exhibited two peaks at ≈1.32 and 1.87 Å, and no peak at 2.65 Å (Figure 1e), indicating the absence of W‐W bond. The former can be assigned to the atomic W coordinated with N, and the latter can be assigned to the W‐P in WP NPs. Best‐fit results extracted from the EXAFS spectra indicated that the coordination number of W‐N in g‐C_3_N_4_/WP/W SAs was 1.7 (Figure S4 and Table S1, Supporting Information). Additionally, wavelet transform (WT) at the W K‐edge EXAFS analysis was further conducted to confirm the atomic dispersion of W atoms in g‐C_3_N_4_/WP/W SAs (Figure 1f,g). Meanwhile, the SEM image and elemental mapping images showed that the elements were distributed on g‐C_3_N_4_/WP/W SAs (Figure S5, Supporting Information). Moreover, the SEM images of g‐C_3_N_4_ (Figure S6a, Supporting Information) showed sheet morphology. After loading with WP/W SAs, the morphology of the samples was similar to that of g‐C_3_N_4_ (Figure S6c,d, Supporting Information). The SEM images of g‐C_3_N_4_/WP showed a sheet‐like morphology (Figure S7, Supporting Information). Compared to g‐C_3_N_4_/WP, g‐C_3_N_4_/WP W SAs have a porous structure and thinner nanosheets (Figure S5, Supporting Information). Moreover, the AFM images of g‐C_3_N_4_/WP/W SAs were consistent with the porous and ultrathin nanosheet in the SEM and TEM images. The thickness of g‐C_3_N_4_/WP/W SAs was 1.2 nm (Figure S8a,b, Supporting Information). Furthermore, the AFM images of WP/W SAs and WP were 2.2 and 29.4 nm, respectively (Figure S8c–f, Supporting Information), suggesting that the vacuum freeze‐drying strategy can effectively combat the van der Waals restacking of the flakes, and generate porous and ultrathin nanosheets.^[^ ^37^, ^38^ ^]^ The loading of W was determined to be 3.05 wt.% by using inductively coupled plasma mass spectrometry (ICP‐MS).

X‐ray diffraction (XRD) indicated the presence of g‐C_3_N_4_ and WP structures in the composite catalyst (Figure S9a, Supporting Information). The structure of samples was examined using FTIR spectra. As shown in Figure S9b (Supporting Information), no noticeable difference can be observed from the FTIR spectra of g‐C_3_N_4_/WP/W SAs, g‐C_3_N_4_/WP, and g‐C_3_N_4_, indicating that the structure of g‐C_3_N_4_ was well preserved after the introduction of WP/W SAs. The interfacial interactions between g‐C_3_N_4_ and WP/W SAs were further investigated by electron paramagnetic resonance (EPR) spectroscopy. As shown in Figure S9c (Supporting Information), a strong signal at the Lorentzian line with g = 2.0038 in the samples can be assigned to an unpaired electron at sp^2^ carbons in π‐conjugated aromatic rings.^[^ ^39^ ^]^ Compared with g‐C_3_N_4_, the EPR signal intensity of g‐C_3_N_4_/WP and g‐C_3_N_4_/WP/W SAs was significantly increased, indicating that the introduction of WP and W SAs into the planar structure of g‐C_3_N_4_ can improve the unpaired electrons and promote charge delocalization, which was beneficial for enhancing the photocatalytic performance.^[^ ^40^, ^41^ ^]^ The signal of g‐C_3_N_4_/WP/W SAs was higher than that of g‐C_3_N_4_/WP due to the formation of numerous unsaturated atoms.^[^ ^42^ ^]^ As shown in Figure S9d (Supporting Information), the I_D_/I_G_ of g‐C_3_N_4_/WP was higher than that of g‐C_3_N_4_/WP/W SAs, suggesting that the electron transfer capability of g‐C_3_N_4_/WP/W SAs was stronger than that of g‐C_3_N_4_/WP because the G band was responsible for the electron conductivity.^[^ ^43^, ^44^ ^]^ In addition, g‐C_3_N_4_ showed no signal (Figure S10, Supporting Information).

The N_2_ adsorption/desorption isotherms shown in Figure S11a (Supporting Information) presented the specific surface areas of photocatalysts. The specific surface area of g‐C_3_N_4_/WP/W SAs was approximately twofold that of g‐C_3_N_4_, illustrating that the introduction of WP/W SAs could increase the specific surface area of photocatalytic active sites in the reaction system. Moreover, g‐C_3_N_4_/WP/W SAs possessed a much higher surface area (172.34 m^2^ g^−1^) than g‐C_3_N_4_/WP (136.98 m^2^ g^−1^) owing to the porosity introduced during the freeze‐drying step used in the synthesis of WP/W SAs.^[^ ^34^ ^]^ As shown in Figure S11b and Table S2 (Supporting Information), the average pore diameters of g‐C_3_N_4_/WP/W SAs, g‐C_3_N_4_/WP and g‐C_3_N_4_ were 5, 3.8, and 10.8 nm, respectively, which can ensure the migration of water molecules.^[^ ^45^ ^]^ g‐C_3_N_4_/WP and g‐C_3_N_4_/WP/W SAs exhibited similar two‐stage decomposition steps (Figure S11c,d, Supporting Information), indicating that the structure of WP was retained during the freeze‐drying process.

X‐ray photoelectron spectroscopy (XPS) was used to determine the chemical composition and to detect the interfacial electronic interaction. The survey XPS spectrum of g‐C_3_N_4_/WP and g‐C_3_N_4_/WP/W SAs in Figure S12 (Supporting Information) displayed the elements of C, N, P, and W. As shown in Figure S13a (Supporting Information), the XPS spectrum of C 1s showed significant changes in the peak intensity and binding energy of g‐C_3_N_4_/WP and g‐C_3_N_4_/WP/W SAs compared to g‐C_3_N_4_, confirming the presence of carbon in g‐C_3_N_4_/WP and g‐C_3_N_4_/WP/W SAs. The high‐resolution C 1s XPS peaks located at 284.8, 286.1, and 289.2 eV could be assigned to C═C/C‐C, C‐O, and C‐N, respectively.^[^ ^45^ ^]^ As shown in Figure S13b (Supporting Information), the N 1s peaks of g‐C_3_N_4_/WP/W SAs were deconvoluted into three peaks at 399.6, 401.2, and 402.2 eV, corresponding to pyridinic N, pyrrolic N, and graphitic N, respectively.^[^ ^46^ ^]^ The N 1s peaks of g‐C_3_N_4_/WP/W SAs exhibited the higher binding energy compared to g‐C_3_N_4_/WP, indicating the formation of an electronic metal‐support interaction. In the P 2p spectrum of g‐C_3_N_4_/WP/W SAs (Figure S14a, Supporting Information), three peaks were observed at 129.9, 130.7, and 133.7 eV, corresponding to the P 2p_3/2_ and P 2p_1/2_ of W‐P bonding, and P‐O bonding.^[^ ^47^ ^]^ The binding energy of P 2p in g‐C_3_N_4_/WP/W SAs exhibited a positive shift relative to WP/W SAs. Consequently, the presence of W SAs caused the charge redistribution between W to P atoms. As shown in Figure S14b (Supporting Information), the W 4f spectrum of g‐C_3_N_4_/WP/W SAs can be fitted into four peaks at 37.86, 35.63, 34.09, and 31.93 eV. The peaks at 31.93 and 34.09 eV of g‐C_3_N_4_/WP/W SAs were assigned to W 4f_7/2_ and W 4f_5/2_.^[^ ^48^ ^]^ Notably, the binding energy of W in the g‐C_3_N_4_/WP/W SAs was slightly shifted to the positive position compared with that of g‐C_3_N_4_/WP, suggesting that the surface electron of W was redistributed in g‐C_3_N_4_/WP/W SAs. Besides, the peaks at 35.63 and 37.94 eV were assigned to the W‐O bond.

### 2.2 Charge Separation Properties

**Figure 2:**
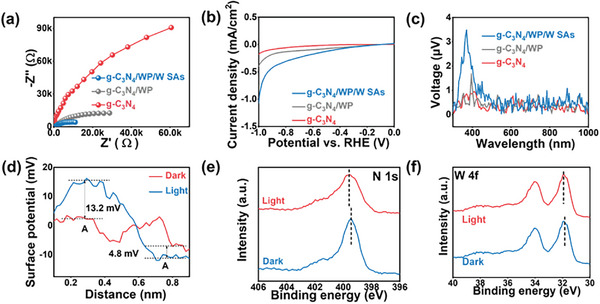
a) Electrochemical impedance spectra (EIS) curves. b) Linear Scan Voltammetry (LSV) curves and c) the SPV potential difference of g‐C_3_N_4_, g‐C_3_N_4_/WP, and g‐C_3_N_4_/WP/W SAs. d) Surface photovoltage under dark and light irradiation of g‐C_3_N_4_/WP/W SAs. High‐resolution XPS spectra for e) N 1s and f) W 4f of g‐C_3_N_4_/WP/W SAs in the dark as well as under simulated sunlight irradiation.

The transient photocurrent response was developed to further evaluate the photogenerated carrier density and charge separation. As shown in Figure S15 (Supporting Information), the photocurrent intensity of g‐C_3_N_4_/WP/W SAs was higher than that of g‐C_3_N_4_ and g‐C_3_N_4_/WP, indicating that the WP and W SAs reduced the recombination of electrons and holes. Furthermore, the arc radius of g‐C_3_N_4_/WP/W SAs was the smallest (**Figure** **2**a), manifesting the much lower interfacial charge transfer resistance in g‐C_3_N_4_/WP/W SAs.^[^ ^49^, ^50^ ^]^ Moreover, the hydrogen evolution potential of g‐C_3_N_4_/WP/W SAs was the lowest among the samples (Figure 2b), suggesting that g‐C_3_N_4_/WP/W SAs was favorable for H_2_ production.^[^ ^46^, ^51^ ^]^ In addition, the SPV was an advanced technique for studying charge separation and transfer processes in photocatalytic systems.^[^ ^52^ ^]^ As presented in Figure 2c, g‐C_3_N_4_ showed a weak SPV response in the region of 300–500 nm, while the SPV response of g‐C_3_N_4_/WP/W SAs was significantly enhanced, indicating that the atomically dispersed W sites remarkably increased the surface potential by constructing an interfacial electric field for the transfer of interfacial charges. Furthermore, according to the Kanada model, the built‐in electric field had a high‐positive correlation with the Zeta potential.^[^ ^53^ ^]^ As shown in Figure S16 (Supporting Information), the Zeta potential of g‐C_3_N_4_/WP/W SAs was −30 mV, while the Zeta potential of g‐C_3_N_4_ was −13.2 mV. This result suggested the presence of strong built‐in electric field in the g‐C_3_N_4_/WP/W SAs composites.

The Kelvin probe force microscopy (KPFM) was used to explore the charge carrier transfer at the interface of g‐C_3_N_4_/WP/W SAs (Figure 2d; Figure S17, Supporting Information). The KPFM surface potential images of g‐C_3_N_4_/WP/W SAs under both dark and simulated sunlight irradiation showed that the surface potential declined by 4.8 mV at position A and increased by 13.2 mV at position B after illumination, implying the transfer of electrons from g‐C_3_N_4_ to WP/W SAs. The in situ XPS tests were also conducted to reveal the electron transfer pathway on the surface of photocatalysts. There was an obvious positive shift (by 0.1 eV) in the N 1s binding energy under light conditions (Figure 2e), suggesting the decreased electron density of N under light irradiation. However, the peaks in W 4f spectra showed a negative shift of 0.04 eV under light irradiation (Figure 2f), suggesting an increase in the electron density of W. Moreover, the C 1s and P 2p peaks for g‐C_3_N_4_/WP/W SAs were shifted to the high binding energy direction under light irradiation as compared to those in the dark (Figure S18, Supporting Information). On account of this, we bring forward the following electron transfer mechanism on the surface of the g‐C_3_N_4_/WP/W SAs. The photogenerated electrons in g‐C_3_N_4_ are further transferred from WP NPs to the W SAs. The single‐particle photoluminescence (PL) spectrum was a strategy to investigate the separation efficiency of electron–hole pairs in photocatalysts (Figure S19, Supporting Information). As shown in Figure S19a (Supporting Information), the g‐C_3_N_4_/WP/W SAs showed the lowest PL emission peak at the wavelength of 487 nm, suggesting that the g‐C_3_N_4_/WP/W SAs possessed a low charge carrier recombination rate. Furthermore, the extended lifetime of g‐C_3_N_4_/WP/W SAs further confirms that WP/W SAs can enhance the interfacial electronic metal‐support interaction (Figure S19b, Supporting Information). The above results demonstrate that W SAs and WP NPs can synergistically enhance the separation of photo‐generated carriers.

### 2.3 The Photothermal Reforming of Plastics to H_2_

**Figure 3:**
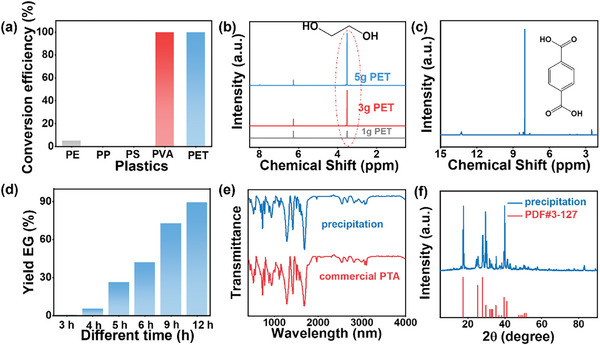
a) The conversion efficiency of different plastics. The concentration of plastics was 0.1g/mL. ^1^H‐NMR spectra of PET pretreatment b) solution and c) precipitation. d) The yield of EG compared to the theoretical value. e) FT‐IR spectra of precipitation and commercial PTA. f) XRD pattern of precipitation.

Previous reports illustrated that pretreatment of PET in alkaline solution formed EG and PTA. Subsequently, EG in the mixture can be selectively oxidized to high value by photoreforming of PET.^[^ ^54^, ^55^, ^56^, ^57^ ^]^ However, the PTA in the mixture does not participate in the catalytic reactions and cannot be directly separated, resulting in material waste. Here, we adopt a strategy of hydrothermal liquefaction pretreatment of PET without alkali to achieve complete separation of EG (supernatant) and PTA (precipitation). Hydrothermal liquefaction, a thermochemical depolymerization process commonly used for liquid fuels production from synthetic and biopolymers, could be used as an alternative approach to treat textile wastes in order to produce value‐added monomeric products. PET plastics can be quantitatively depolymerized into their monomers PTA and EG in sub and supercritical water.^[^ ^58^ ^]^ As shown in **Figure** **3**a and Figure S20 (Supporting Information), different types of plastics were hydrolyzed under subcritical water conditions. PE, PP, and PS hardly changed after hydrothermal liquefaction, indicating the high stability of C‐C. Notably, PET with C‐O bonds exhibited a conversion rate close to 100%. ^1^H‐NMR confirmed that the supernatant and precipitation were indeed EG and PTA, respectively (Figure 3b,c). As shown in Figure S21 (Supporting Information), ^13^C‐NMR confirmed that the precipitation was PTA. In addition, Figure S22 (Supporting Information) showed that there was no significant difference between the supernatant and commercial EG, indicating that the supernatant was EG. Moreover, the quality of EG was determined for different hydrothermal treatment time, respectively (Figure 3d; Figure S23, Supporting Information). The results were basically similar to the theoretical value of PET hydrolysis (1.35 g EG in 3 g PET), indicating that the hydrothermal liquefaction condition can fully achieve the hydrolysis of PET plastics and have higher efficiency than the alkaline solution at room temperature.^[^ ^15^ ^]^ Furthermore, the precipitation obtained after drying were tested by XRD pattern and FT‐IR (Figure 3e,f), and the results suggested that the product was indeed PTA. The reasons for achieving the separation of EG and PTA are as follows: 1) the high temperature provides energy to break C‐O bonds in plastics; 2) the generated PTA is slightly soluble in water and exhibits acidity at high temperature; 3) most of the PTA can precipitate in the Teflon‐lined autoclave under the cooling environment and acidic conditions.

**Figure 4:**
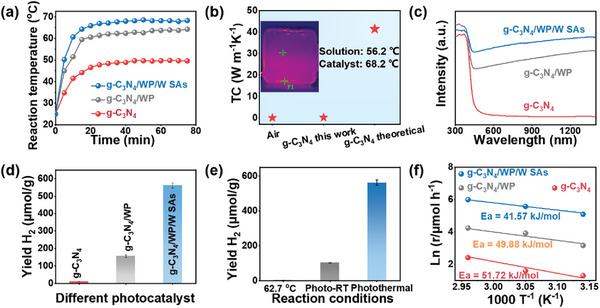
a) The solution temperature profiles of g‐C_3_N_4_, g‐C_3_N_4_/WP, and g‐C_3_N_4_/WP/W SAs dispersed in solution. b) Comparison of the thermal conductivity of g‐C_3_N_4_ in this work with other reported samples. Inset: IR thermal images of g‐C_3_N_4_/WP/W SAs. c) UV–vis diffuse reflectance spectra of g‐C_3_N_4_, g‐C_3_N_4_/WP, g‐C_3_N_4_/WP/W SAs and WP/W SAs. d) Photothermal reforming of PET using different samples without temperature control. e) Photoreforming (room temperature, 25 °C), thermal reforming (62.7 °C without light irradiation), and photothermal reforming of PET to H_2_ over g‐C_3_N_4_/WP/W SAs. f) Apparent activation energy of the reaction system at different temperatures of g‐C_3_N_4_, g‐C_3_N_4_/WP and g‐C_3_N_4_/WP/W SAs.

In a typical procedure, 10 mg of the photocatalyst was dispersed in 5 mL of PET supernatant after hydrothermal treatment under continuous stirring. The photothermal reforming of PET to H_2_ over the as‐synthesized photocatalysts was investigated under simulated sunlight light using solution after hydrothermal treatment of PET as a sacrificial reagent. Digital infrared thermal imaging (DITI) was used to acquire the center infrared thermal images. The temperature change of the solution containing g‐C_3_N_4_, g‐C_3_N_4_/WP, g‐C_3_N_4_/WP/W SAs in the quartz reactor without any thermal insulation under illumination was measured (Figures S24 and S25, Supporting Information; **Figure** **4**a). The solution temperature of g‐C_3_N_4_/WP/W SAs and g‐C_3_N_4_/WP rapidly increased from 50.2 to 68.1 °C and 44.7 to 62.4 °C, respectively, while g‐C_3_N_4_ increased from 41.7 to 48.9 °C, indicating that WP NPs can induce photothermal effects and enhance the local surface temperature. Interestingly, the thermal conductivity of g‐C_3_N_4_ (0.04253 W m^−1^K^−1^) was much lower than the theoretical thermal conductivity (Figure 4b), indicating that the g‐C_3_N_4_ can suppress heat loss to the surroundings. The porous g‐C_3_N_4_ showed a relatively low thermal conductivity and similar to that of air (0.0267 W m^−1^ K^−1^), confirming the presence of a considerable amount of pore structures inside the samples.^[^ ^59^, ^60^ ^]^ In addition, the inset photographs showed that the temperature of the g‐C_3_N_4_/WP/W SAs was higher than that of the solution, indicating that g‐C_3_N_4_ can act as an insulating layer to inhibit heat loss. As shown in Figure 4c, the UV–vis diffuse reflectance spectra (DRS) showed that WP NPs enhanced the light absorption in the 400–800 nm range. The corresponding band gaps (E_g_) of g‐C_3_N_4_/WP, g‐C_3_N_4_/WP/W SAs and WP/W SAs were 2.02, 2.03, and 2.70 eV, respectively (Figure S26, Supporting Information). According to the color photographs of the samples, the color of the g‐C_3_N_4_/WP/W SAs was darker compared to g‐C_3_N_4_/WP (Figure S27, Supporting Information), indicating that the introduction of W SAs can induce stronger photothermal effects.

Next, the performance of the photocatalysts was evaluated in the photothermal reforming of PET by monitoring the amount of H_2_ evolution. As shown in Figure 4d, the trace amounts of H_2_ were generated over g‐C_3_N_4_. After decorating WP on g‐C_3_N_4_, the g‐C_3_N_4_/WP can increase the activity, suggesting that the P atom with strong electronegativity can attract electrons from the W atom. Then, the negatively charged P can act as a base to capture positively charged protons during the reaction process (Figure S28, Supporting Information). Interestingly, the coexistence of W SAs and WP NPs in the g‐C_3_N_4_/WP/W SAs further enhanced the reaction rate, suggesting the synergistic effect of WP NPs and W SAs. The photothermal reforming of PET over g‐C_3_N_4_ with different amounts of WP/W SAs is shown in Figure S29 (Supporting Information). The highest amount of hydrogen evolution was 562.5 µmol h^−1^ g^−1^ over g‐C_3_N_4_/WP/W SAs (presented g‐C_3_N_4_/WP‐3/W SAs), ≈56 times higher than that of g‐C_3_N_4_. These results suggested that the addition of WP/W SAs could significantly improve the photocatalytic ability of g‐C_3_N_4_ and excessive amount of WP/W SAs could reduce the photocatalytic activity. Notably, no detectable H_2_ was observed over g‐C_3_N_4_/WP/W SAs in the absence of sacrificial agent, illumination or photocatalyst, confirming that the light source was necessary (Figure S30, Supporting Information). To exclude the role of C in the photothermal reforming of PET, the hydrogen evolution of g‐C_3_N_4_/PC was much lower than that of g‐C_3_N_4_/WP/W SAs (Figure S31, Supporting Information), indicating that the C had no photocatalytic activity. It was found that different temperatures of hydrothermal pretreatment of PET affected the performance (Figure S32, Supporting Information). The possible reason was that the amount of EG formed was different within the temperature range. Similarly, the activity of different amounts of PET was also tested (Figure S33, Supporting Information). When the amount of PET was 3 g, the hydrogen evolution rate was 562.5 µmol h^−1^ g^−1^. In conclusion, the amount of EG from PET did indeed affect the performance of photothermal reforming of PET to H_2_ within a certain range.

To investigate the photothermal effect of photocatalyst, the hydrogen activities of g‐C_3_N_4_/WP/W SAs were tested within 2.5 h of irradiation under different conditions (Figure 4e). The photothermal catalytic activity (562.5 µmol h^−1^ g^−1^) was higher than the photocatalytic activity (101.5 µmol h^−1^ g^−1^). To illustrate the thermal effect mechanism of g‐C_3_N_4_/WP/W SAs, thermal catalytic hydrogen evolution under different temperatures was systematically employed. As shown in Figure S34 (Supporting Information), the thermal catalytic hydrogen evolution of all samples was improved with the increase of temperature in the dark. The hydrogen evolution of WP/W SAs was improved compared to that of g‐C_3_N_4_ and g‐C_3_N_4_/WP/W SAs, confirming that the synergistic effect of WP NPs and W SAs as a thermal catalyst induced lattice heating under temperature‐driven. According to the previous temperature curve, WP was mainly responsible for photothermal conversion. The increased temperature was used for the activation of H‐OH by electron‐phonon coupling.^[^ ^12^ ^]^ According to the Arrhenius formula (supporting information), the apparent activation energy (*E*_a_) was obtained by fitting the hydrogen evolution rate and reaction temperature (Figure 4f). Notably, the *E*_a_ of g‐C_3_N_4_/WP/W SAs was significantly reduced to 41.57 kJ mol^−1^ compared with that of pristine g‐C_3_N_4_ (51.72 kJ mol^−1^), indicating that the photothermal effect induced by WP/W SAs significantly accelerated the rate‐limiting step of photo‐thermal reforming of PET to H_2_. Moreover, the hydrogen evolution rate exhibited a regular linear relationship with increasing light intensity (Figure S35, Supporting Information), suggesting that WP/W SAs can accelerate the migration of photogenerated carriers at the interface to improve the efficiency of hydrogen production.^[^ ^12^, ^61^ ^]^ In addition, the cycling tests were performed to evaluate the stability of the catalyst during photothermal reforming of PET (Figure S36, Supporting Information). The cycling tests of g‐C_3_N_4_/WP/W SAs under simulated sunlight irradiation showed no significant performance degradation during 3 runs of tests. The SEM images, XRD patterns and FTIR spectra (Figures S37 and S38, Supporting Information) of g‐C_3_N_4_/WP/W SAs before and after reaction were almost identical, indicating the stability of g‐C_3_N_4_/WP/W SAs.

### 2.4 Mechanism of the Photothermal Reforming of Plastic to H_2_

**Figure 5:**
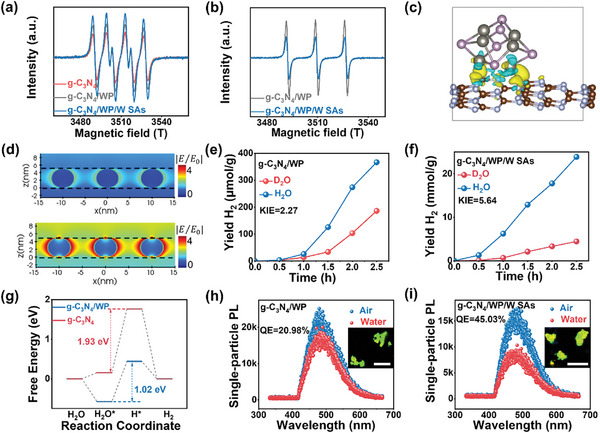
a) EPR signals of DMPO‐•O_2_^−^ for g‐C_3_N_4_/WP, g‐C_3_N_4_/WP/W SAs, and g‐C_3_N_4_. b) TEMPO‐h^+^ of g‐C_3_N_4_/WP and g‐C_3_N_4_/WP/W SAs. c) Calculated differential charge distribution of g‐C_3_N_4_/WP. d) Finite‐difference time‐domain (FDTD) result of g‐C_3_N_4_/WP at the wavelength of 400 nm (top part) and 700 nm (lower part). Measured KIE over e) g‐C_3_N_4_/WP and f) g‐C_3_N_4_/WP/W SAs. g) Free energy diagram of g‐C_3_N_4_ and g‐C_3_N_4_/WP. Single‐particle PL spectra of h) g‐C_3_N_4_/WP and i) g‐C_3_N_4_/WP/W SAs immersed in air and water. Inset: single‐particle PL lifetime mappings of g‐C_3_N_4_/WP and g‐C_3_N_4_/WP/W SAs, respectively, QE stands for quenching efficiency.

g‐C_3_N_4_/WP/W SAs displayed strong signals of superoxide radicals (•O_2_^−^) (**Figure** **5**a), which sufficiently confirmed the synergistic effect of W SAs and WP NPs. In contrast to g‐C_3_N_4_, g‐C_3_N_4_/WP/W SAs exhibited lower TEMPO‐h^+^ signal (TEMPO: 2, 2, 6, 6‐tetramethylpiperidinooxy) under irradiation for 5 min (Figure 5b), confirming the enhanced ability of photoexcited electron‐hole pair separation by decorating atomically dispersed W SAs and WP NPs as cocatalysts.^[^ ^62^ ^]^ According to the calculated average potential profile along the z‐axis of g‐C_3_N_4_ and WP, their work functions were calculated to be 4.42 and 4.9 eV, respectively (Figure S39a,b, Supporting Information). Theoretically, the electrons of g‐C_3_N_4_ should spontaneously transfer to WP at the interface of the heterojunction, and the *E*_F_ of WP would rise up to match the *E*_F_ of g‐C_3_N_4_. In addition, the differential charge density diagrams (Figure 5c) of g‐C_3_N_4_/WP and g‐C_3_N_4_ were presented. At the heterojunction interface, the yellow surface represents strong charge accumulation and blue surface stands for obvious charge depletion. At the interface, the electrons were concentrated on the surface of WP, while the electron density at the g‐C_3_N_4_ surface was decreased. Visibly, the charge redistribution mainly occurred at the g‐C_3_N_4_/WP interface region, where there was an intense electron interaction.^[^ ^63^ ^]^ Thus, the charge redistribution established up an electric field from g‐C_3_N_4_ to WP at the heterojunction interface.

To illustrate the promoting effect of photothermal effect on carrier transport in the built‐in electric field of g‐C_3_N_4_ and WP, 3D‐FDTD simulation was employed to determine the spatial distribution of electric field intensity of g‐C_3_N_4_/WP under 400 and 700 nm monochromatic light excitation (Figure 5d). It is evident that the overall electromagnetic field enhancement (|E|/|E_0_|) of g‐C_3_N_4_/WP under 700 nm excitation was much stronger than that under 400 nm light excitation, due to the presence of the built‐in electric field at the interface and the interfacial electron enrichment effect by photothermal effect.^[^ ^61^ ^]^ Meanwhile, the near‐field enhancement can promote the activation of reaction molecules, thereby improving the catalytic activity.^[^ ^64^, ^65^ ^]^ DFT calculation further investigated the field enhancement effect on H_2_O adsorption and H_2_ desorption. Figure S40a,b (Supporting Information) showed the optimized structures of H_2_O and H_2_ on g‐C_3_N_4_/WP with an electric field of 0.05 V Å^−1^. Conspicuously, the adsorption energy of H_2_O adsorbed on g‐C_3_N_4_/WP with an electric field of 0.05 V Å^−1^ was more negative than that with no electric field (Figure S40c, Supporting Information), suggesting that the electric field played an important role in H_2_O activation. Interestingly, the adsorption energy of H_2_ adsorbed on g‐C_3_N_4_/WP was more positive at an electric field of 0.05 V Å^−1^ compared with that no electric field, indicating that the electric field enhanced H_2_ desorption.

The kinetic isotope effect (KIE) was an effective strategy to confirm the rate‐limiting step (RDS).^[^ ^66^ ^]^ As shown in Figure 5e, g‐C_3_N_4_/WP exhibited much lower hydrogen evolution when D_2_O was used as the solvent, and the value of the KIE was calculated to be 2.27. As shown in Figure 5f, the KIE of g‐C_3_N_4_/WP/W SAs was much larger than that of g‐C_3_N_4_/WP, suggesting that W SAs was responsible for the cleavage of the O‐H bonds. In addition, the activation of reactant molecules can be enhanced by the larger pore size and specific surface area of g‐C_3_N_4_/WP/W SAs. Moreover, it also suggested that the source of H_2_ was from H_2_O rather than the PET substrate, which was consistent with other reports.^[^ ^67^ ^]^ By calculating the Gibbs free energy change of the reaction on g‐C_3_N_4_ (Figure 5g), the largest increase in free energy (rate‐limiting step) was the step of water dissociation (1.93 eV), which was water in adsorbed state (*H_2_O) got electron and was reduced to hydrogen proton (*H). After loading with WP and W SAs, the free energy barrier of the rate‐limiting step was dropped to 1.02 and 0.54 eV (Figure S41, Supporting Information), indicating that WP and W single atoms can synergistically activate the H‐OH bond of absorbed water by reducing the apparent activation energy of water spilling. The single‐particle PL spectra were an effective tool to investigate the water activation on catalysts,^[^ ^66^ ^]^ as excited photocatalysts were highly sensitive to the surrounding environment during energy/charge transfer.^[^ ^68^, ^69^ ^]^ The quenching efficiencies of g‐C_3_N_4_, g‐C_3_N_4_/WP and g‐C_3_N_4_/WP/W SAs were 3.16%, 20.98% and 45.03%, respectively (Figure 5h,i; Figure S42, Supporting Information), suggesting the activation of H_2_O molecules by W SAs. In addition, these results revealed charge transfer from g‐C_3_N_4_/WP/W SAs to water molecule. Based on the above characterizations and analysis, the proposed mechanisms for g‐C_3_N_4_/WP/W SAs in the photothermal reforming of PET to H_2_ can be described as follows. Under light irradiation, the electron–hole pairs were generated in the surface of g‐C_3_N_4_. Then, the WP/W SAs could efficiently capture the photoexcited electrons from the g‐C_3_N_4_ and the interface of WP/W SAs can act as the electron‐rich sites for reducing H_2_O to H_2_. The interface of g‐C_3_N_4_ would act as the hole‐rich sites for the oxidation of PET to glyoxal and glycolate (Figure S43, Supporting Information).

## 3 Conclusion

In summary, g‐C_3_N_4_/WP/W SAs was successfully designed for the photothermal reforming of plastics and exhibited promising H_2_ evolution performance (562.5 µmol g_cat_^−1^ h^−1^). The high activity was attributed to three aspects: (1) The WP‐induced photothermal effect can improve the slow kinetics of hydrogen evolution in the photo‐reforming of PET and reduce the apparent activation energy. (2) W SAs can serve as reactive sites for water activation and promote O‐H cleavage. (3) g‐C_3_N_4_ not only produces photogenerated carriers but also exerts heat preservation, which together ensure the excellent catalytic performance. Moreover, alkali‐free hydrothermal pretreatment of plastics was reported, which was a simple and environmentally friendly strategy for the plastic pretreatment. Significantly, an obvious PL quenching phenomenon of g‐C_3_N_4_/WP/W SAs was observed when in contact with water molecules, implying the transfer of photogenerated electrons to H_2_O. This work not only provides a novel understanding of the details of photothermal reforming of plastics, but also provides theoretical guidance for the rational design of photothermal catalysts to achieve efficient H_2_ generation from plastics.

## Conflict of Interest

The authors declare no conflict of interest.

## Supporting information



Supporting Information

## Data Availability

Research data are not shared.
