# Supplementary material for: Reinforcing the Efficiency of Plastic Upgrading through Full‐Spectrum Photothermal Effect Integration of Heat Isolator
Source: Adv Sci (Weinh). 2024 Oct 28;11(48):2410260. doi: 10.1002/advs.202410260 (PMC11672297; doi:10.1002/advs.202410260)
Supplement: Supplementary file 1 — Supporting Information [file ADVS-11-2410260-s001.docx]

**Supporting Information**

**Reinforcing the Efficiency of Plastic Upgrading through Full-Spectrum Photothermal Effect Integration of Heat Isolator**

*Xueqin Gong, Peng Wang, Shuo Yang, Wenbo Li, Min Lv, Bei Li, Xiangxiang Zhang, Zeyan Wang, Yuanyuan Liu, Peng Wang, Hefeng Cheng, Ying Dai, Baibiao Huang and Zhaoke Zheng**

X. Gong, W. Li, M. Lv, B. Li, X. Zhang, Z. Wang, Y. Liu, P. Wang, H. Cheng, B. Huang and Z. Zheng

State Key Laboratory of Crystal Materials,

Shandong University

Jinan 250100, China

Email: [zkzheng@sdu.edu.cn](mailto:zkzheng@sdu.edu.cn)

Y. Dai

School of Physics,

Shandong University,

Jinan 250100, China

P. Wang and S. Yang

School of Chemistry and Chemical Engineering,

Shandong University,

Jinan 250100, China

**Chemicals and reagents.**

All chemicals and solvents were purchased from commercial suppliers and can be used without further purification. Urea was purchased from Sinopharm Chemical Reagent Co. Melamine was purchased from Aladdin Biochemical Technology Co., Ltd.

Phytic acid and Ammonium metatungstate hydrate ((NH_4_)_6_H_2_W_12_O_40_·xH_2_O) were purchased from Macklin. Poly-(ethylene terepththalate) was obtained from Bide Pharmatech Ltd.

**Activation energy calculations:** The E_a_ was calculated by using the Arrhenius equation^[1]^:

ln𝑘=𝐶−𝐸_𝑎_/𝑅𝑇

Where k was conversion rate of hydrogen, R= 8.314 J mol^–1^ K^–1^ was the gas constant, T was reaction temperature.

**Characterizations.**

The phase and crystal structure of as-synthesized samples were confirmed by X-ray diffraction (XRD) using a Bruker AXS D8 diffractometer with Cu Kα radiation (λ = 1.5406 Å and operation conditions with 40 kV and 100 mA) in the range from 5 to 90° (2θ). The morphology of synthesized samples was investigated by scanning electron microscopy (SEM) (Hitachi S-4800), transmission electron microscopy (TEM) (JEM-F200) and high-angle-annular-dark-field scanning transmission electron microscopy (HAADF-STEM) (Tatin Themis G2). Ultraviolet-visible (UV-vis) spectra were taken on a Shimadzu (UV-3600 plus) spectrophotometer using BaSO_4_ as reference sample. X-ray photoelectron spectroscopy (XPS) survey spectra was used to investigate the element compositions and states on Thermo SCIENTIFIC ESCALAB Xi+ with a monochromic Al Kα X-ray source. The in situ XPS measurements were performed at Thermo SCIENTIFIC ESCALAB 250Xi. Thermogravimetric (TG) analyses were performed on a Mettler-TGA/DSC 3+ thermal analyzer with a heating rate of 10 °C min^-1^ in air from 30 °C to 800 °C. The Fourier-transform infrared spectrometer (FT-IR, Bruker Tensor II) was used to measure the functional groups with a KBr disk in the range of 4000-400 cm^-1^. The magnetic properties were measured by compressing the samples into linear diamagnetic plastic capsules (Quantum Design). The elements were examined by ICP spectrometry (Jarrell-Ash, USA). The electron paramagnetic resonance spectra (ESR) of the samples were determined using Bruker-A300 spectrometer. The Raman spectra was obtained on Horiba scientific-LabRAM HR evolution spectrometer using 325 nm as the excitation light source. The Brunauer-Emmett-Teller (BET) specific surface areas of samples were determined by a multipoint BET method, and the pore size distributions were determined using desorption branches through the Barrett-Joyner-Halenda (BJH) method (Builder Kubo X-1000 apparatus). The surface photovoltage (SPV) spectra was performed at CEL-SPS1000. The surface temperature of the catalyst was measured by the thermal imaging camera (HIKMICRO). Surface potential distribution of g-C_3_N_4_/WP/W SAs under dark and λ > 420 nm conditions were measured by the Kelvin probe force microscopy measurements (KPFM) (Bruker multimode 8). The thermal diffusion coefficients of the samples were performed using TPS2500s. The TPD was obtained on China-Xianquan TP5080.

**NMR Measurement**

^1^H NMR and ^13^C NMR analysis were conducted on Bruker Avance III HD 400 at room temperature to characterize the structure. The supernatant (0.5 mL) was diluted in 0.1 mL of 10 mM DMSO (used as internal standard) D_2_O solution. The precipitation was diluted in (CD_3_)_2_S=O (sample: 0.5 mL; (CD_3_)_2_S=O: 0.1 mL).

**FDTD Calculations**

In this paper, the finite element software FDTD was used to calculate the electric field distribution of the nanostructures. We set up periodic boundary conditions in the x, and y-directions, while perfectly matched layer conditions are used in the z-direction. A plane wave was incident vertically along the z-direction onto the surface of the nanostructure as an excitation light source. Importantly, for the accuracy of the computational results, the mesh of the computational region was divided into 0.2 nm × 0.2 nm × 0.2 nm. In addition, its convergence time was set to 1×10^(-5).

**XAS measurements and analysis**

The obtained XAFS data was processed in Athena (version 0.9.26) [J. Synchrotron Rad. 2005, 12, 537] for background, pre-edge line and post-edge line calibrations. Then Fourier transformed fitting was carried out in Artemis (version 0.9.26) [J. Synchrotron Rad. 2005, 12, 537]. The k^2^ weighting, k-range of 3 – ~12.5 Å^-1^ and R range of 1 - 3 Å were used for the fitting of W-foil; The k^2^ weighting, k-range of 3 – ~11 Å^-1^ and R range of 1-2 Å were used for the fitting of Samples.

For Wavelet Transform analysis, the χ(k) exported from Athena was imported into the Hama Fortran code. The parameters were listed as follow: R range, 0-4 Å; k range, 0-16 Å^-1^; k weight, 2; and Morlet function with κ=10, σ=1 was used as the mother wavelet to provide the overall distribution.

**Density functional theory (DFT) calculations.**

The Vienna Ab Initio Package (VASP) was used to perform all density functional theory (DFT) calculations within the generalized gradient approximation (GGA) using the PBE formulation ^[4-7]^. We have chosen the projected augmented wave (PAW) potentials to describe the ionic cores and take valence electrons into account using a plane wave basis set with a kinetic energy cutoff of 400 eV. Partial occupancies of the Kohn−Sham orbitals were allowed using the Gaussian smearing method and a width of 0.05 eV. The electronic energy was considered self-consistent when the energy change was smaller than 10^−5^ eV. A geometry optimization was considered convergent when the force change was smaller than 0.02 eV/Å. Grimme’s DFT-D3 methodology was used to describe the dispersion interactions.

The equilibrium lattice constant of hexagonal g-C_3_N_4_ monolayer 2x2 supercell separated by a vacuum layer in the depth of 20 Å was optimized to be a=13.674 Å. It is used as model 1. Model 2 was built by adding one Ag4 cluster onto model 1. During structural optimizations, the gamma point in the Brillouin zone was used for k-point sampling, and all atoms were allowed to relax.

The adsorption energy (E_ads_) of adsorbate A was defined as

E_ads_ = E_A/surf_ – E_surf_ – E_A(g)_

where E_A/surf_, E_surf_ and E_A(g)_ are the energy of adsorbate A adsorbed on the surface, the energy of clean surface, and the energy of isolated A molecule in a cubic periodic box with a side length of 20 Å and a 1×1×1 Monkhorst-Pack k-point grid for Brillouin zone sampling, respectively.

**Synthesis of g-C_3_N_4_.**

The urea was placed in a porcelain boat, heated to 550 °C with a ramp rate of 0.5 °C·min^-1^, and kept for 3 h in air. Then, 0.1 g of powder was heated to 500 ℃ at a rate of 5 ℃ /min in a muffle furnace for 2 hours. The sample was labeled as g-C_3_N_4_.

**Synthesis of WP/W SAs and WP NPs**

The preparation of WP/W SAs was derived from by thermal polymerization of freeze-dried pretreated precursor. Briefly, the melamine (5g), ammonium metatungstate hydrate (0.3 g) and phytic acid (1 mL) were dispersed in 70 mL of deionized water to form a mixture solution and stirred for 5 h. After that, the mixed solution was freeze-dried to remove deionized water. The obtained powders were heated to 850 °C with a ramp rate of 5 °C·min^-1^ for 2 h. The product was denoted as WP/W SAs. For comparison, the PC was synthesized with similar procedures without the addition of ammonium metatungstate hydrate^[2]^. As a comparison, the WP NPs was prepared through the same procedure without freeze-dry.

**Synthesis of g-C_3_N_4_/WP/W SAs and g-C_3_N_4_/WP.**

40 mg g-C_3_N_4_ and different amount of WP/W SAs (x=8, 24, 60, 120 mg) were added to 40 mL distilled water, respectively. The solution was sonicated for 1 hour and then stirred for 1 hour. Afterward, we centrifuged the suspension to collect the precipitate and then washed it with deionized water several times. Finally, the solution was dried at 80 ℃ for 12 h. The sample was denoted as g-C_3_N_4_/WP-1/W SAs, g-C_3_N_4_/WP-2/W SAs, g-C_3_N_4_/WP-3/W SAs and g-C_3_N_4_/WP-4/W SAs. In addition, g-C_3_N_4_/WP-3/W SAs was the optimal catalyst, which was named g-C_3_N_4_/WP/W SAs. As a comparison, the photocatalyst was obtained by the same procedure with adding PC or WP, which was denoted as g-C_3_N_4_/C and g-C_3_N_4_/WP.

**Hydrothermal pretreatment of PET.**

PET was pretreated by one-step solvothermal method ^[3]^. Different amount (x=1, 3 and 5g) of PET commercial powder was added to 50 mL deionized. Then the mixture was transferred into a 100 mL Teflon-lined autoclave and maintained at 200 °C for 12 h.

**Photoelectrochemical measurement.**

The working photoelectrodes were prepared as follows: 5 mg of photocatalyst powders were dispersed in mixed solution containing 4.5 mL of isopropanol, and 100 µL of Nafion with ultrasound treatment for 30 min. Then, the mixture solution was made into electrode (fluorine-doped tin oxide (FTO) glass substrate, 1 × 1 cm squares) by drop casting method and dried in oven at 100 °C for 1 h. Photoelectrochemical measurements were performed by using an electrochemical workstation (CHI 760D) and carried out in a standard three-electrode quartz cell with a platinum wire as the counter electrode, an Ag/AgCl electrode as the reference electrode and 0.5 M Na_2_SO_4_ aqueous solution as the electrolyte. A 300 W Xenon light with an AM 1.5 simulated sunlight was utilized as the light source.

**Photothermal experiments on photothermal reforming of PET to H_2_.**

Typically, 10 mg photocatalyst was dispersed in 5 mL hydrothermal pretreatment of PET solution. After the mixed solution was bubbled by Ar for 10 min in the dark, the mixture was irradiated by a 300 W Xe lamp with a full-light irradiation without circulating water for cooling. The real-time temperature was measured and recorded by an infrared thermal imaging thermometer.

**Recycling experiments for photothermal reforming of PET to H_2_**.

Recycling experiments were investigated under the same conditions to test the stability of the photocatalysts. 10 mg of g-C_3_N_4_/WP/W SAs to a 5 mL of pretreated PET solution. Then, the quartz reactor was bubbled with Ar for 10 min to vent air. After 2.5 h of illumination, the yield of H_2_ was also measured. After each test, the photocatalyst was centrifuged and dried at 80 °C to use for the next recycle.

**Single-particle PL measurements.**

The g-C_3_N_4_, g-C_3_N_4_/WP and g-C_3_N_4_/WP/W SAs were separately dispersed in MilliQ ultrapure water. The quartz glass cover was ultrasonically cleaned multiple times with HNO_3_ solution and deionized water. The well-dispersed photocatalysts suspension was spin-coated on a clean quartz cover glass. Single-particle PL images and spectra were performed on a scanning confocal microscope system (PicoQuant, MicroTime 200) combined with an Olympus IX73 inverted fluorescence microscope. The photocatalysts were excited by an oil-immersion objective lens (Olympus, UplanSApochromat, 100×, 1.4 NA) with a 375 nm continuous wave laser controlled by a PDL-800B driver (PicoQuant). The time-resolved PL spectra was obtained with 375 nm excitation, the decay curves were fitted by three-exponential decay function to acquire deconvolution of the instrument response function. More importantly, to simulate the real environment of charge transfer between g-C_3_N_4_/WP/W SAs and H_2_O in the photocatalysis of PET to H_2_, the in-situ single-particle PL spectra was tested by adding different solutions to cover glass coated with the g-C_3_N_4_/WP/W SAs. The single-particle PL spectra of g-C_3_N_4_/WP/W SAs in air was tested. Then, the single-particle PL spectra and single-particle lifetime of g-C_3_N_4_/WP/W SAs in water were obtained by dripping H_2_O onto a quartz cover glass coated with the g-C_3_N_4_/WP/W SAs.


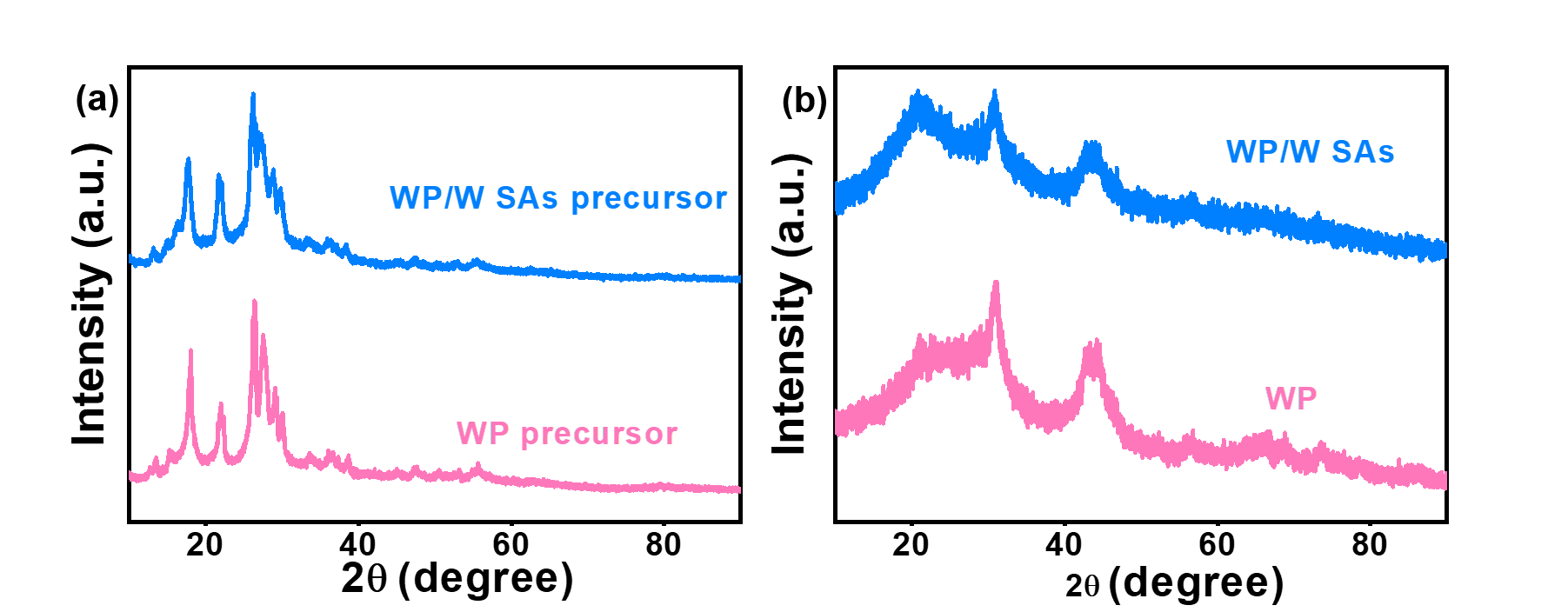


**Figure S1.** (a) XRD patterns of WP/W SAs precursor and WP precursor. (b) XRD patterns of WP/W SAs and WP.


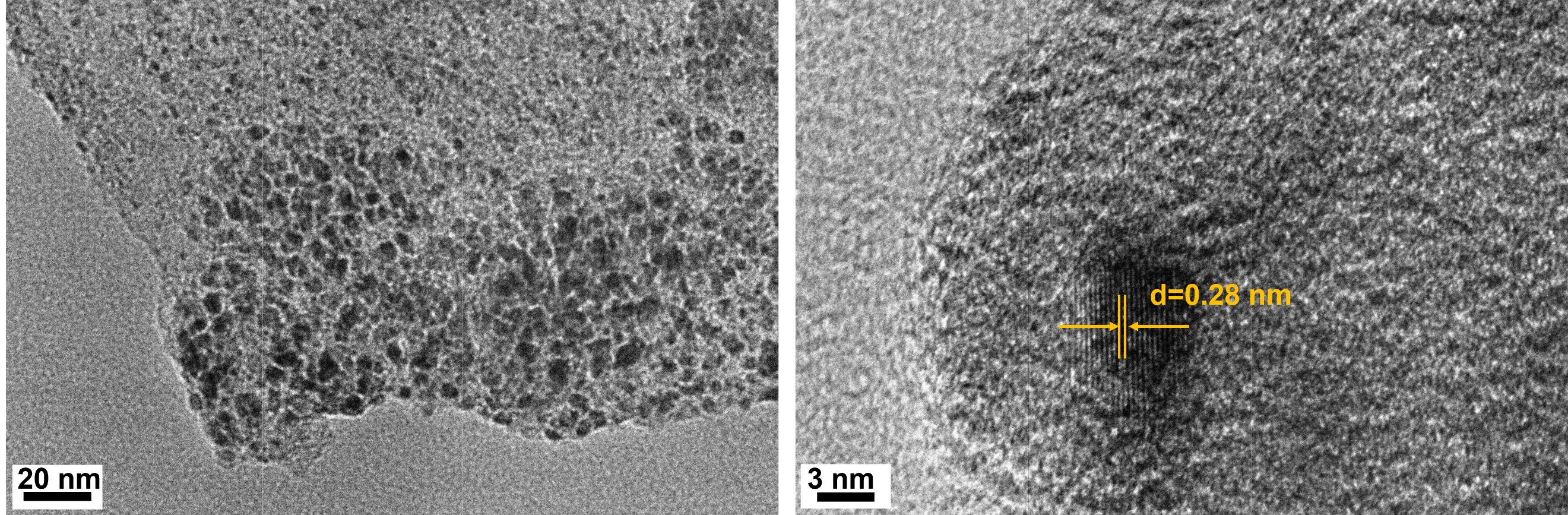


**Figure S2.** TEM images of g-C_3_N_4_/WP.


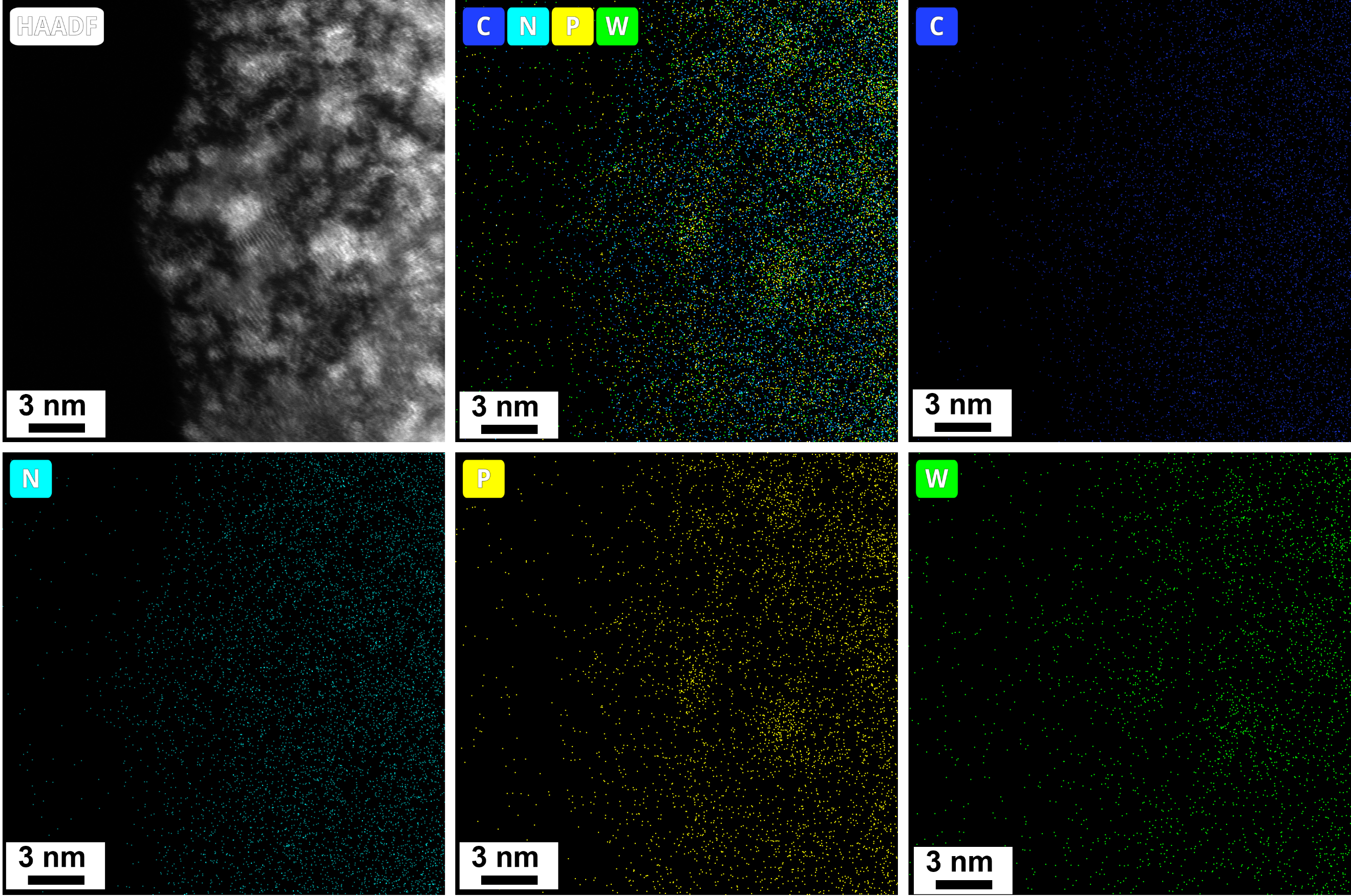


**Figure S3.** HAADF-STEM-EDS mapping of g-C_3_N_4_/WP/W SAs.


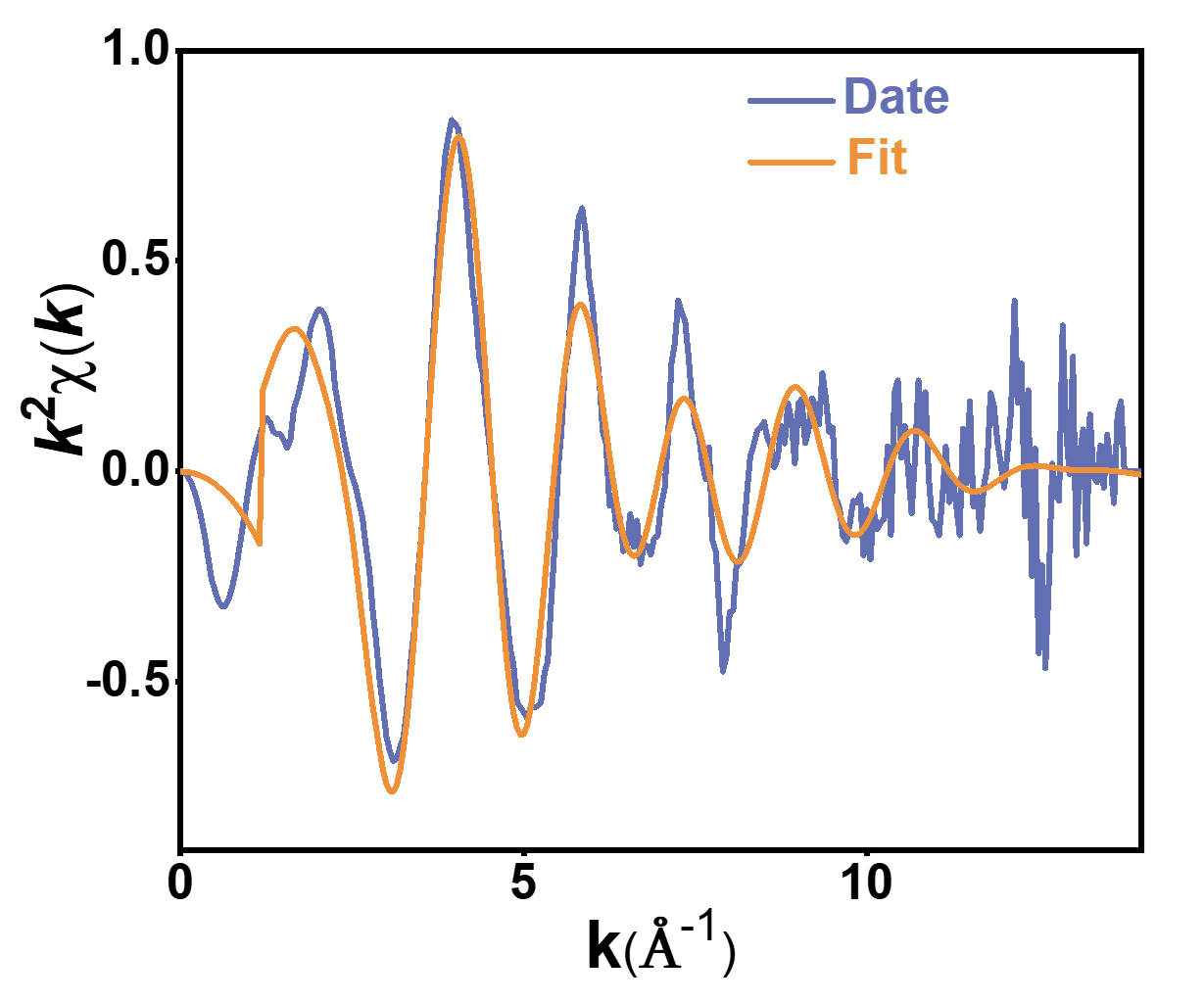


**Figure S4.** EXAFS fitting curve in R space of g-C_3_N_4_/WP/W SAs.


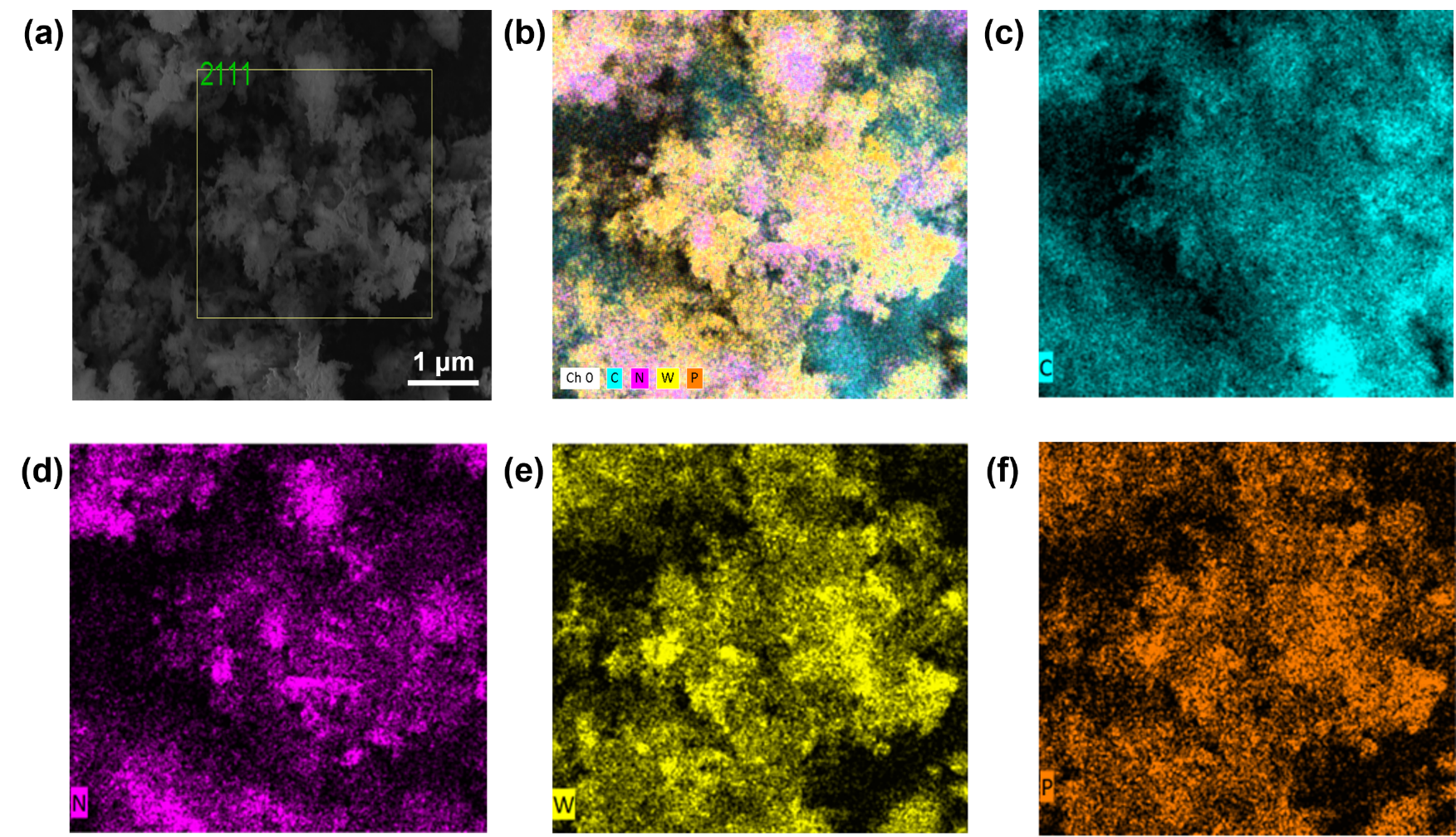


**Figure S5.** (a) SEM image and (b-f) corresponding elemental mapping images of (c) C, (d) N, (e) W and (f) P of g-C_3_N_4_/WP/W SAs.


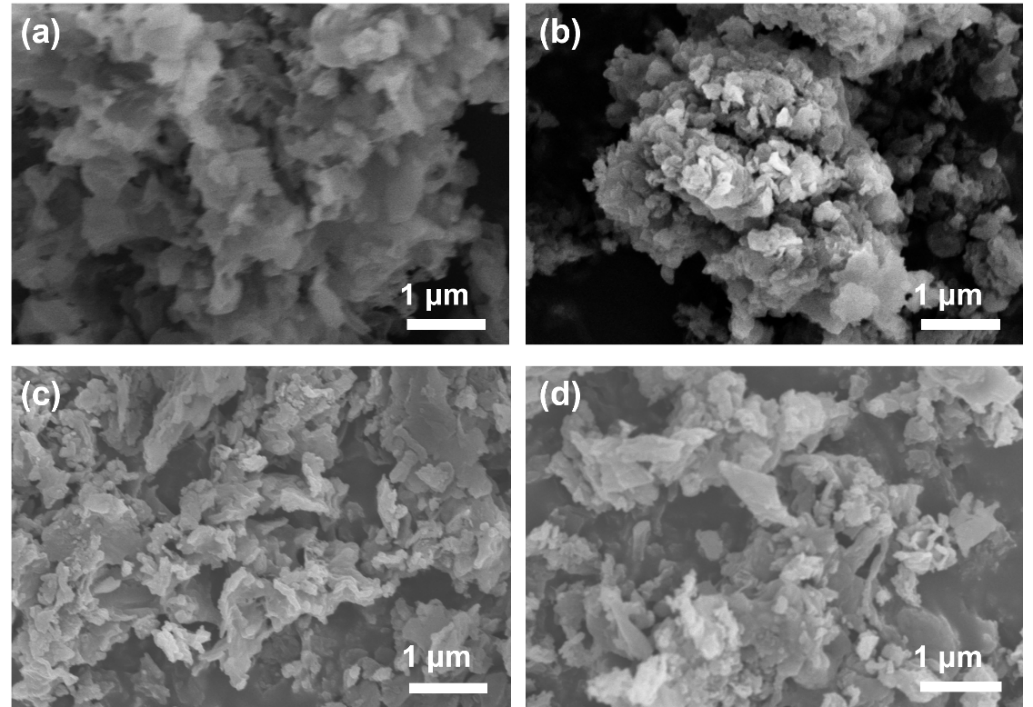


**Figure S6.** SEM images (a) g-C_3_N_4_, (b) g-C_3_N_4_/WP-1/W SAs, (c) g-C_3_N_4_/WP-2/W SAs and (d) g-C_3_N_4_/WP-4/W SAs.


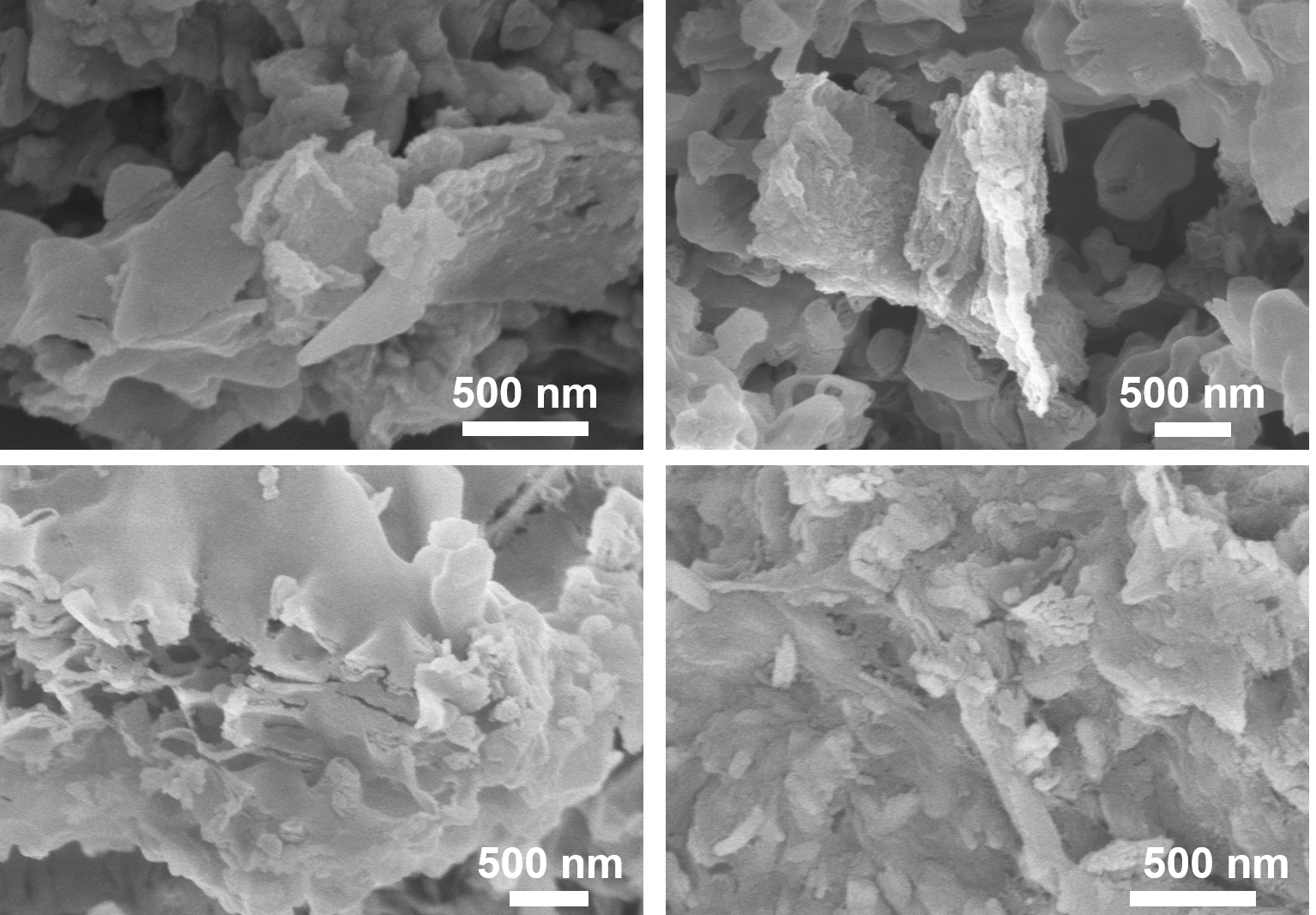


**Figure S7.** SEM images of g-C_3_N_4_/WP.


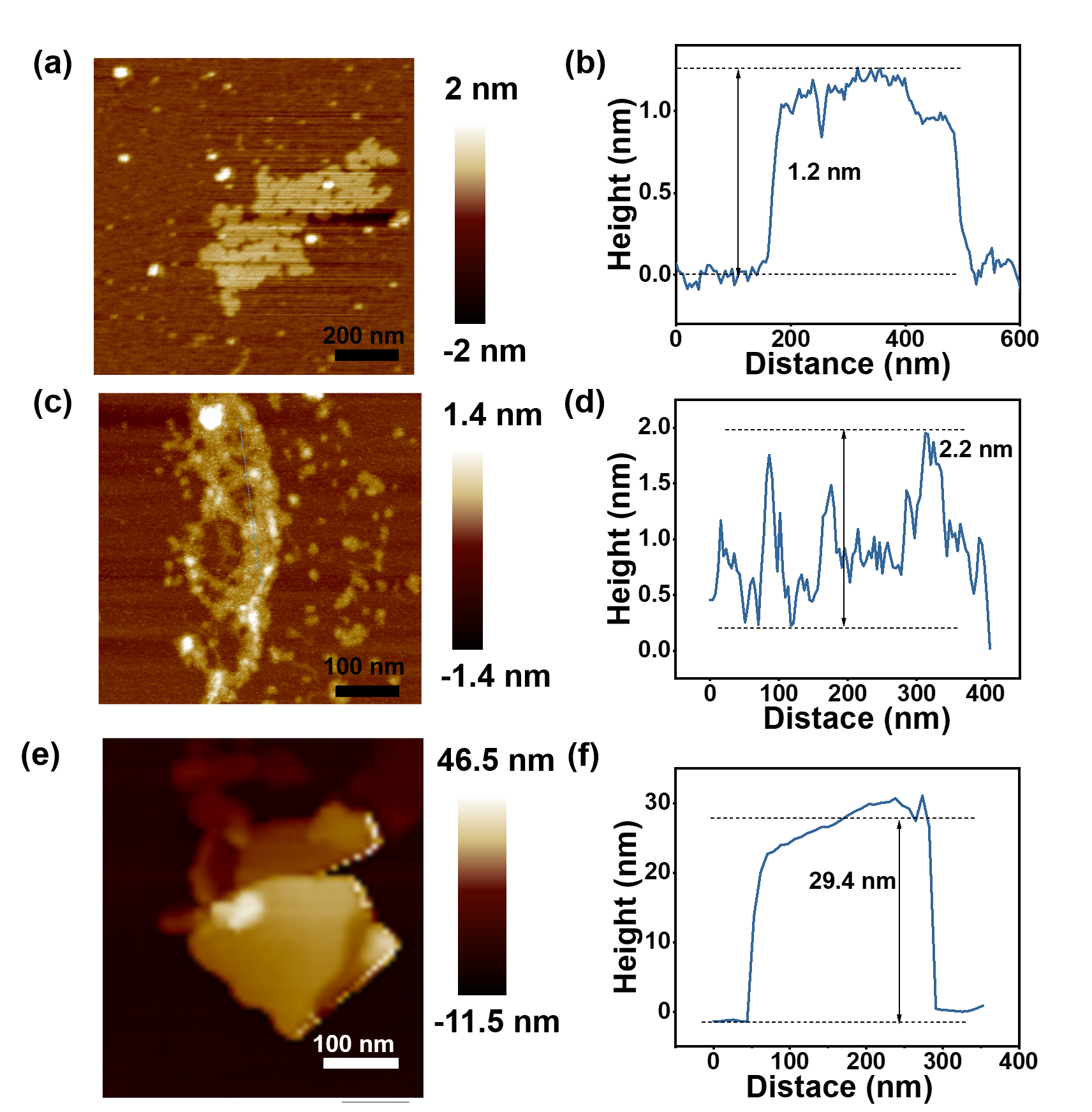


**Figure S8.** (a) AFM image and (b) height profiles of g-C_3_N_4_/WP/W SAs. (c) AFM image and (d) height profiles of WP/W SAs. (e) AFM image and (f) height profiles of WP.


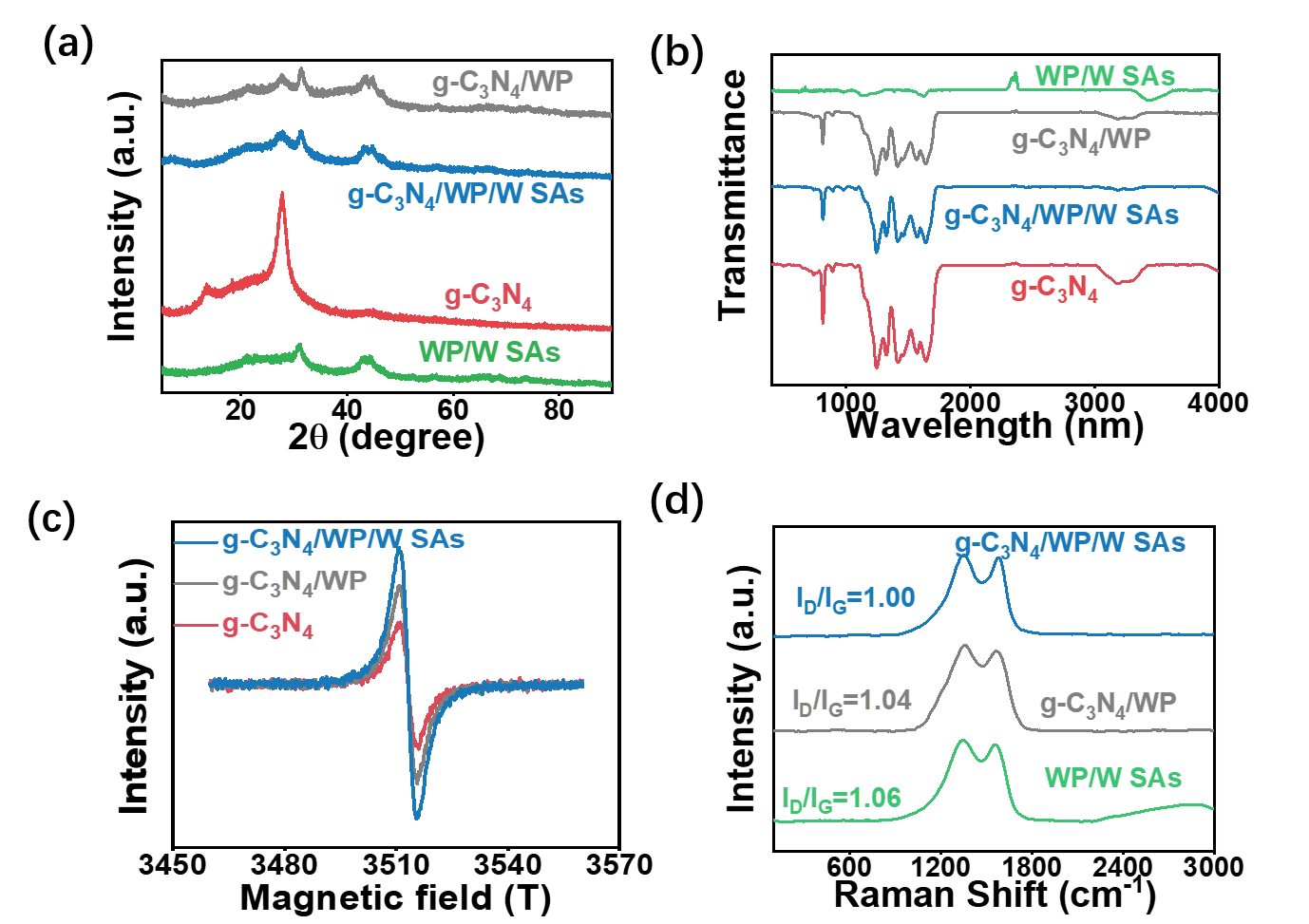


**Figure S9.** (a) XRD patterns of g-C_3_N_4_, WP/W SAs, g-C_3_N_4_/WP/W SAs and g-C_3_N_4_/WP, respectively. (b) FTIR of g-C_3_N_4_, WP/W SAs, g-C_3_N_4_/WP/W SAs and g-C_3_N_4_/WP, respectively. (c) EPR profiles at room temperature of g-C_3_N_4_, g-C_3_N_4_/WP/W SAs and g-C_3_N_4_/WP, respectively. (d) Raman spectrum of WP/W SAs, g-C_3_N_4_/WP/W SAs and g-C_3_N_4_/WP.

The XRD pattern of g-C_3_N_4_/WP/W SAs (Figure S9a) showed peaks at 21.2°, 31.2°, 43.1° and 44.6°, corresponding to the (101), (011), (112) and (211) facets of WP (PDF no. 29-1364)^[2]^. Obviously, the relatively sharp peak located at 27.71° can be assigned to the periodic stacking (002) of g-C_3_N_4_ ^[4]^. WP have been found in g-C_3_N_4_/WP/W SAs and g-C_3_N_4_/WP. As can be clearly seen from Figure S9b, the sharp breathing peak at 809 cm^-1^ of g-C_3_N_4_-based samples was assigned to the triazine units, and the peaks between 1167 and 1683 cm^-1^ was the characteristic feature of g-C_3_N_4_ stretching vibration modes^[5]^. The broad peak within 3000-3600 cm^-1^ was attributed to N-H groups^[6]^. As can be seen from Figure S9d, g-C_3_N_4_/WP/W SAs, g-C_3_N_4_/WP and WP/W SAs showed two peaks corresponding to 1355 and 1578 cm^-1^, which were ascribed to the D and G bands of graphene, respectively ^[7]^. Moreover, the I_D_/I_G_ for WP/W SAs was the highest, as the combination of the WP/W SAs and g-C_3_N_4_ leaded to a decrease in the amount of N-doped graphene^[8]^.


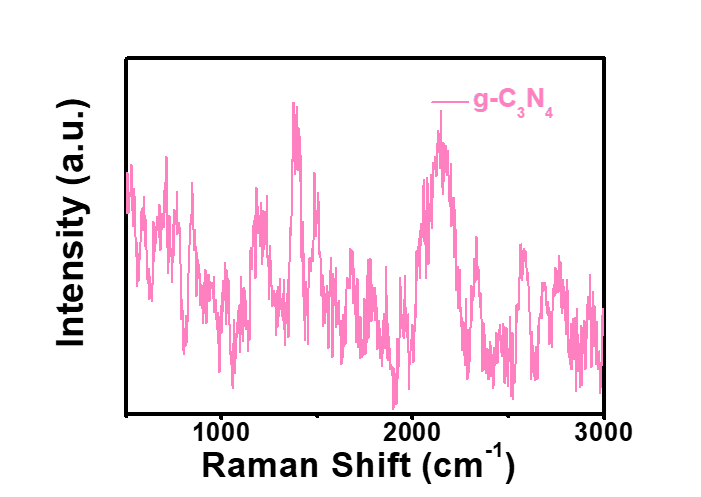


**Figure S10.** Raman spectra of g-C_3_N_4_.

**
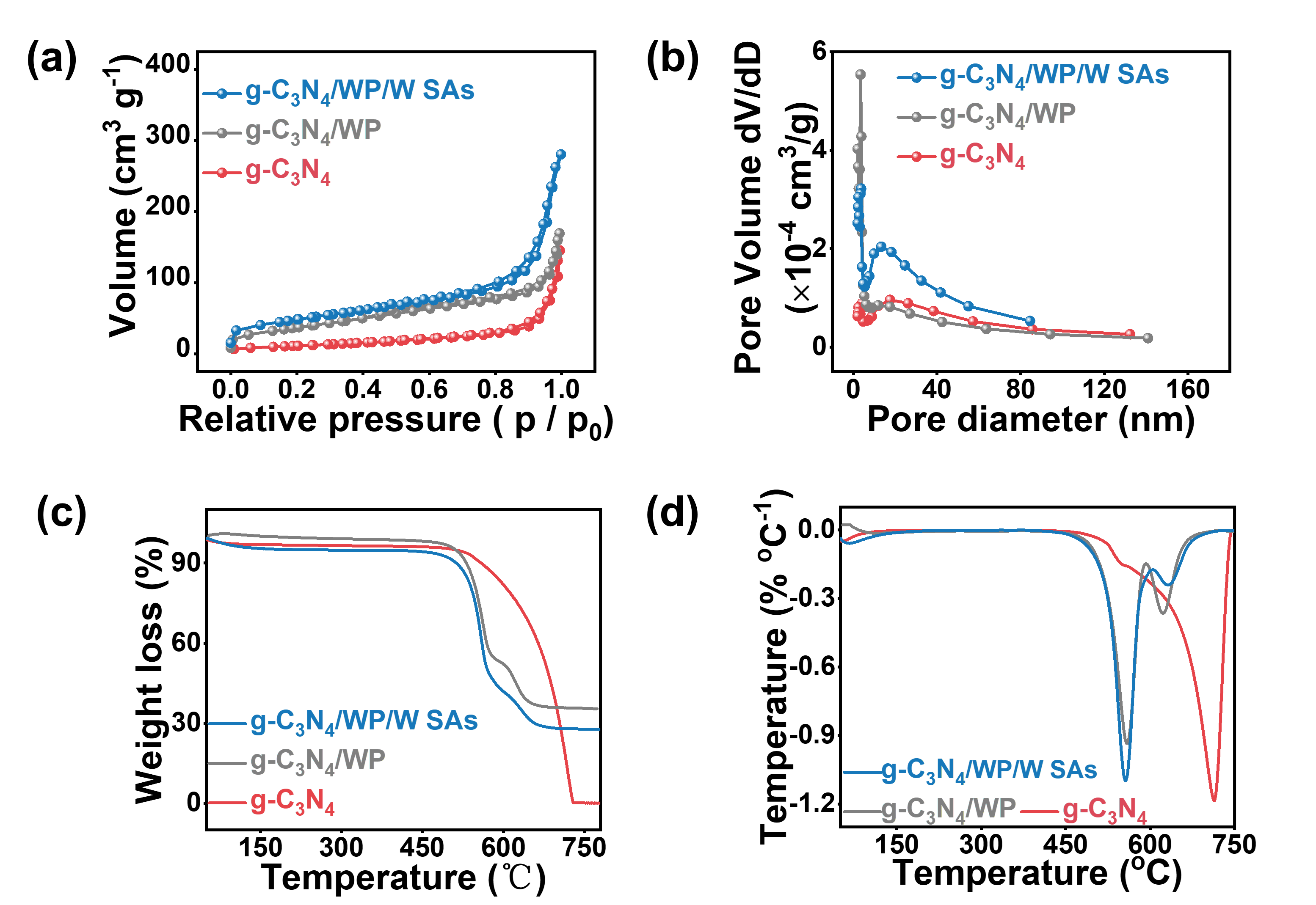
**

**Figure S11.** (a) N_2_ adsorption-desorption isotherms and (b) the corresponding pore size distribution curves of g-C_3_N_4_/WP/W SAs, g-C_3_N_4_/WP and g-C_3_N_4_. (c) TG and (d) DTG thermograms of g-C_3_N_4_/WP/W SAs, g-C_3_N_4_/WP and g-C_3_N_4_.


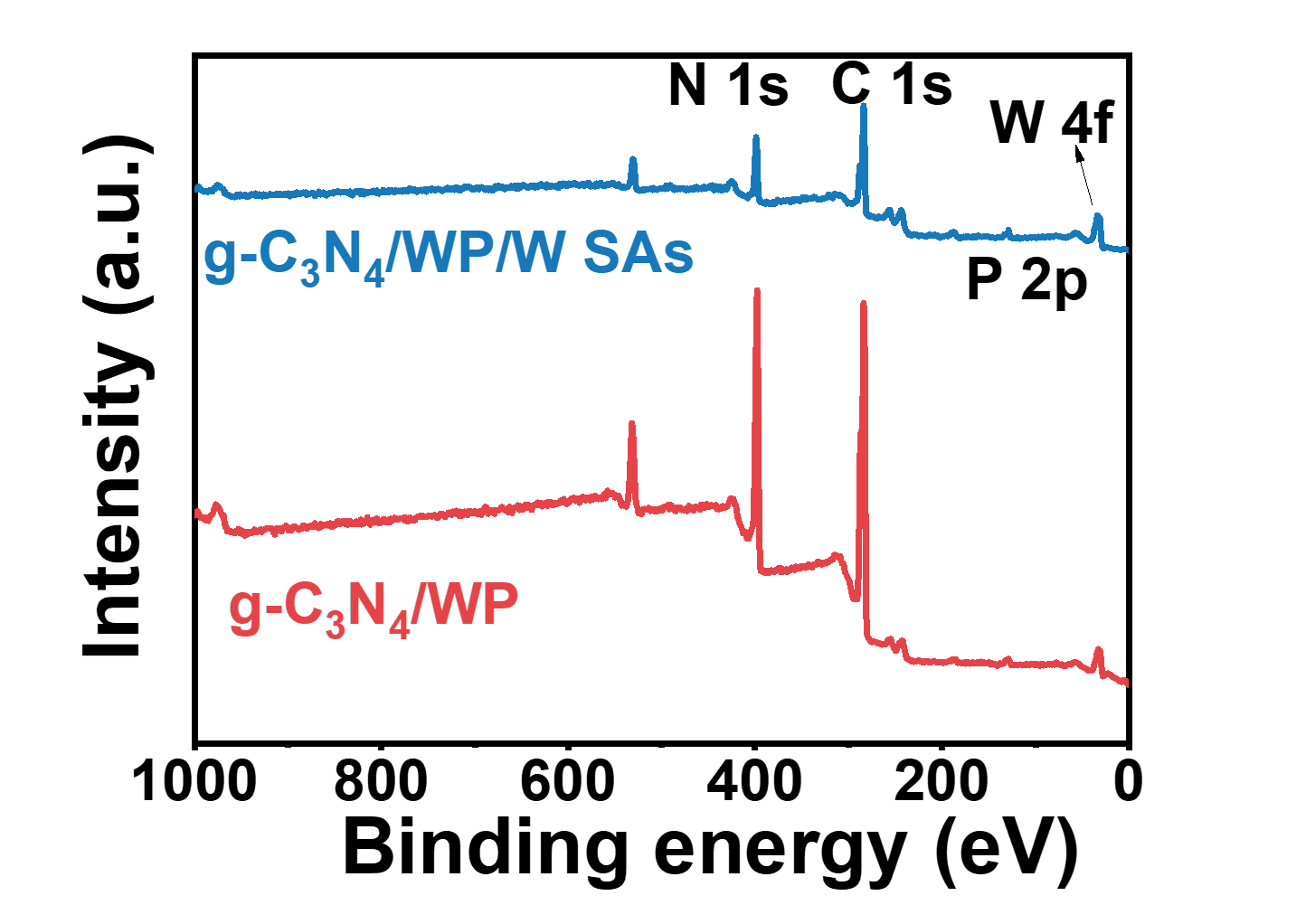


**Figure S12.** XPS spectrum of g-C_3_N_4_/WP/W SAs and g-C_3_N_4_/WP.


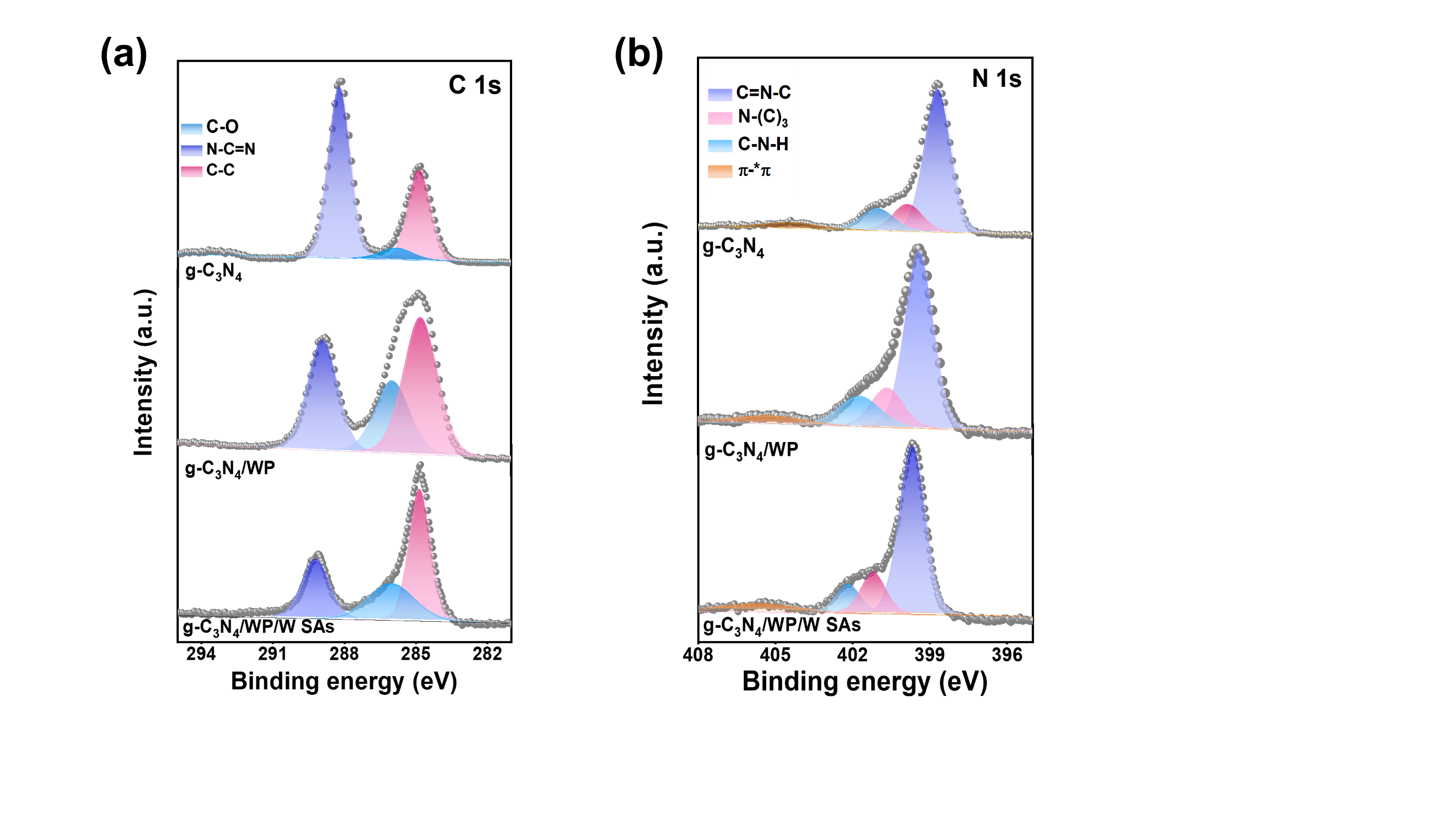


**Figure S13.** High resolution XPS spectra in the region of (a) C 1s and (b) N 1s of g-C_3_N_4_/WP/W SAs, g-C_3_N_4_/WP and g-C_3_N_4_.


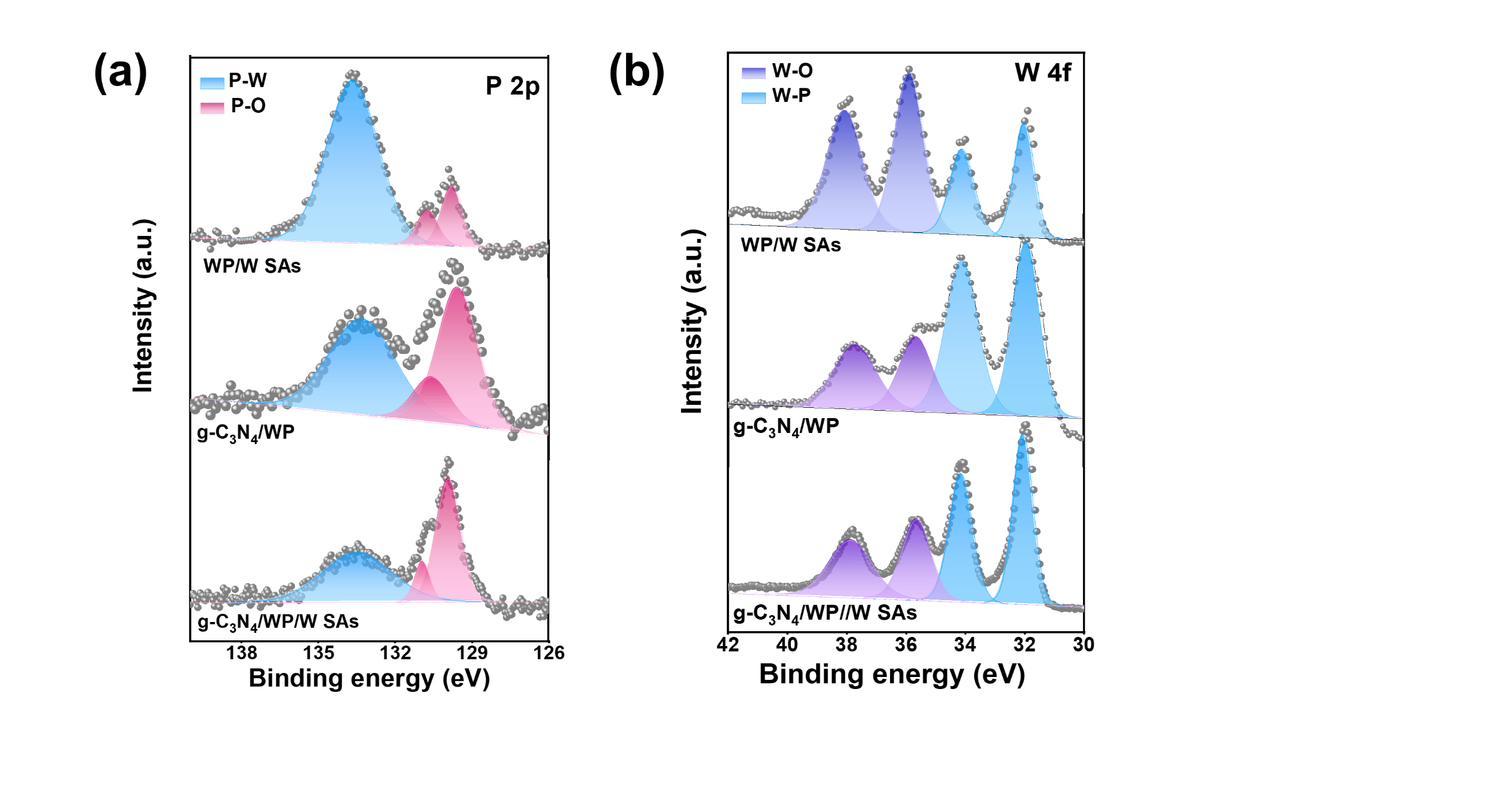


**Figure S14.** (a) P 2p and (b) W 4f of g-C_3_N_4_/WP/W SAs, g-C_3_N_4_/WP and WP/W SAs.


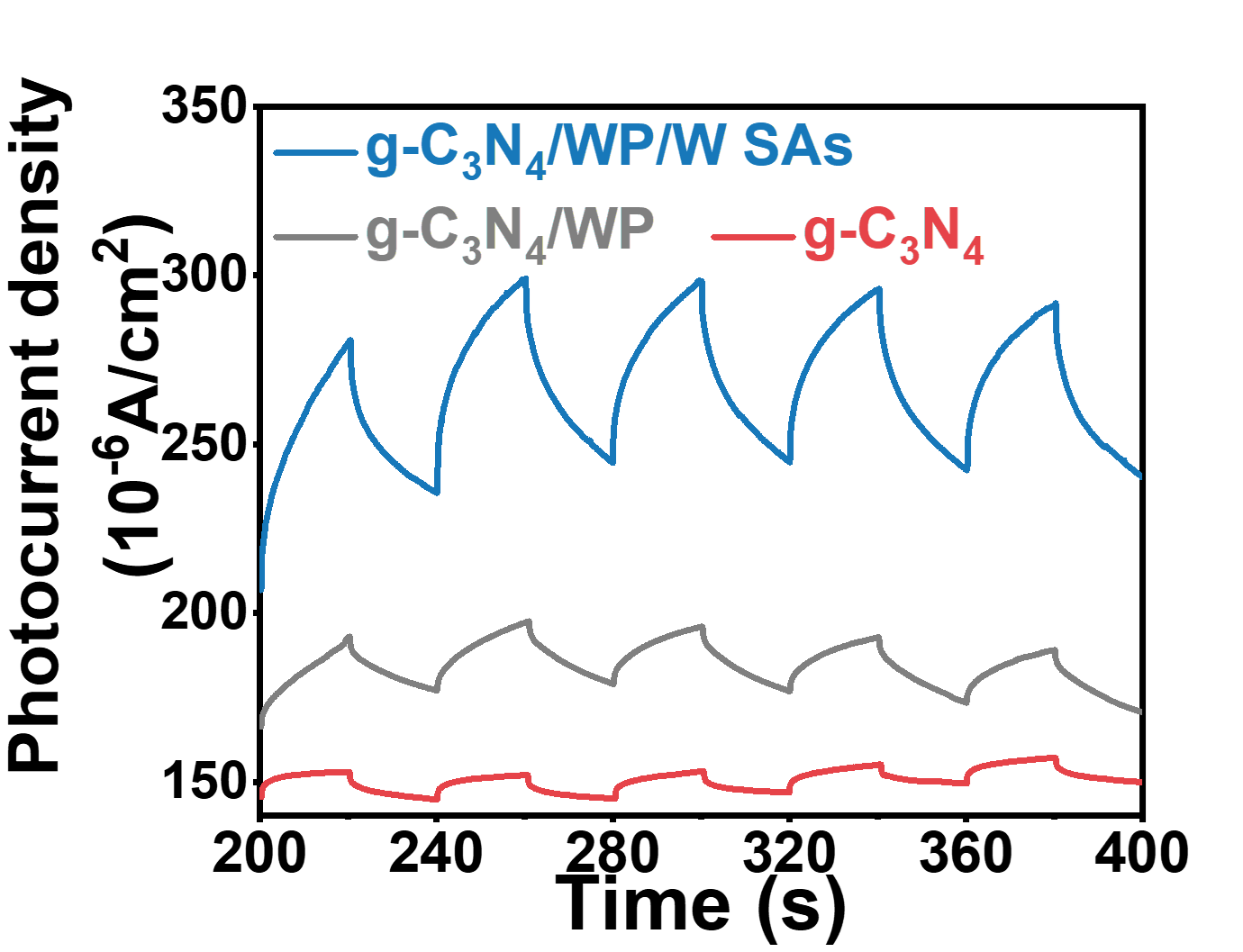


**Figure S15.** Transient photocurrent density-time plots of g-C_3_N_4_/WP/W SAs, g-C_3_N_4_/WP and g-C_3_N_4_.


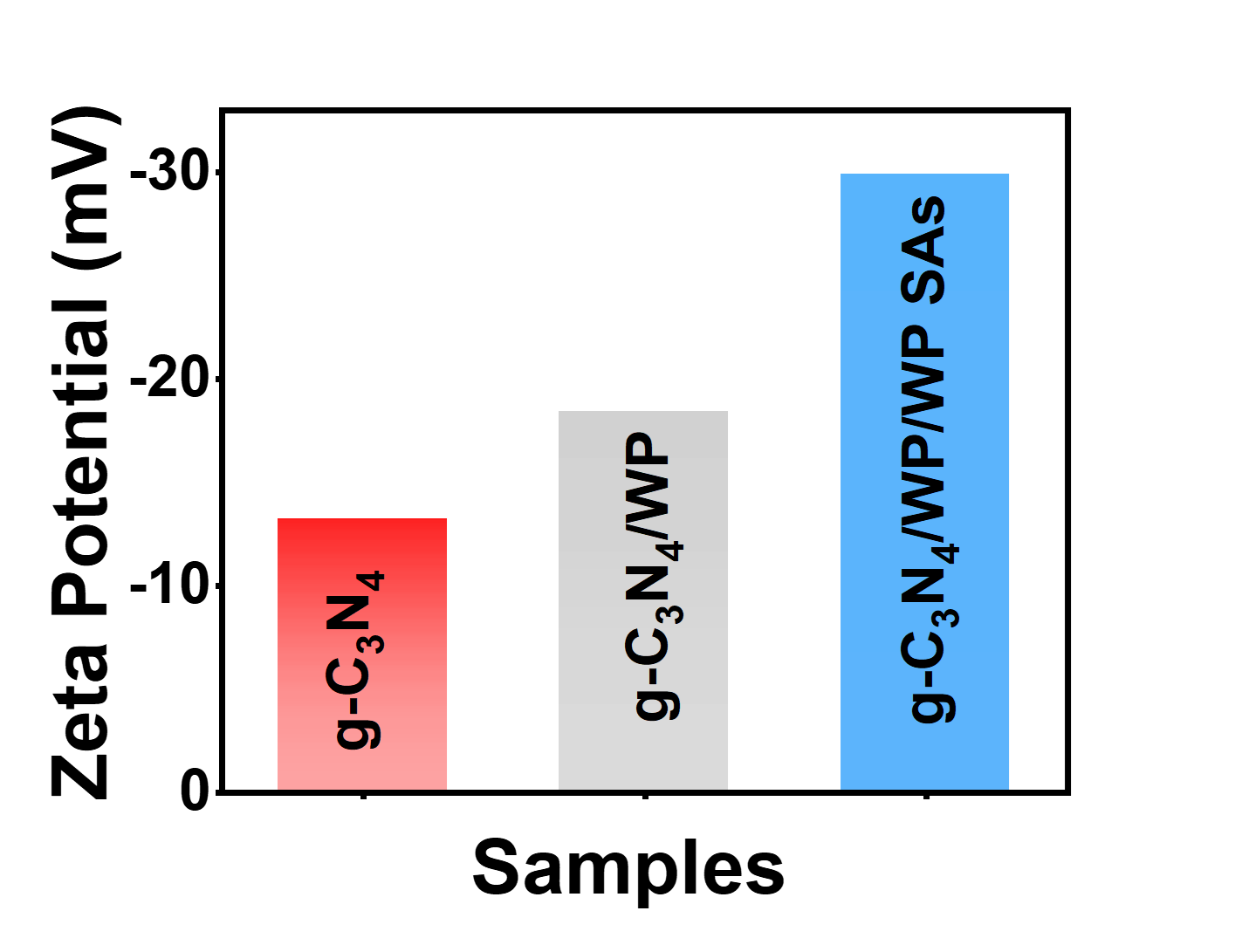


**Figure S16.** Zeta potential of g-C_3_N_4_/WP/W SAs, g-C_3_N_4_/WP and g-C_3_N_4_.


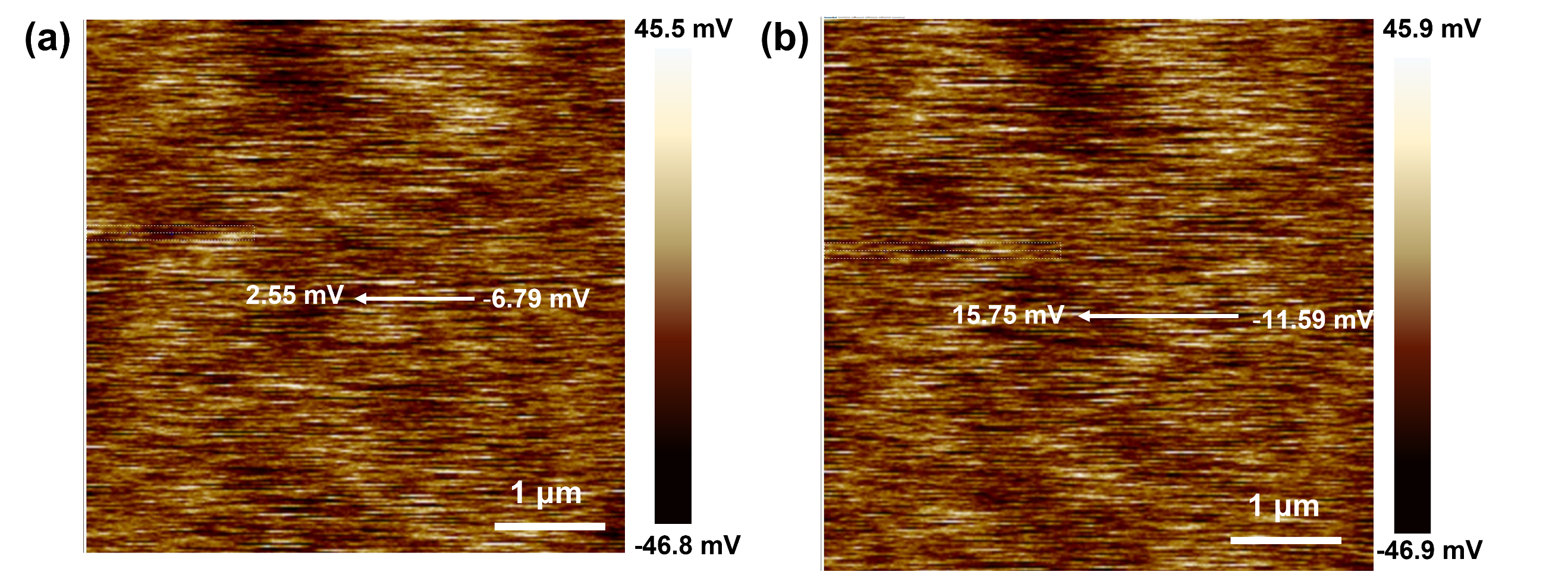


**Figure S17.** Surface photovoltage distribution of g-C_3_N_4_/WP/W SAs under (a) dark and (b) light irradiation conditions.


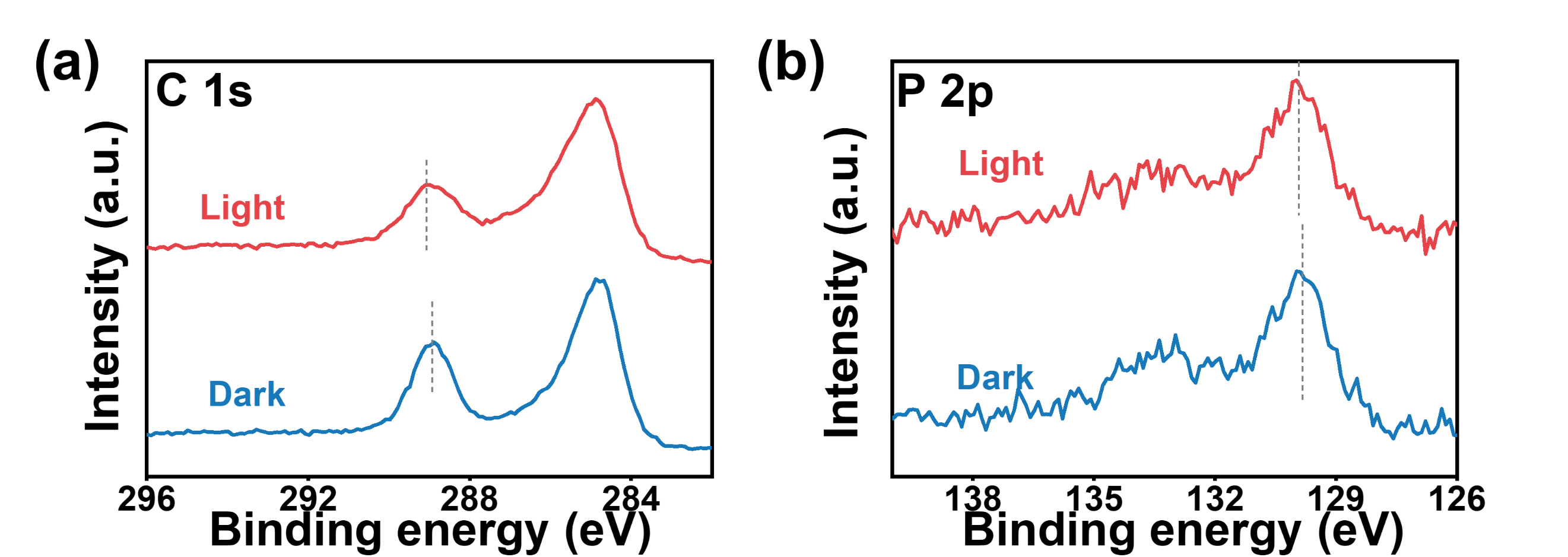


**Figure S18.** High-resolution XPS spectra for (a) C 1s and (b) P 2p of g-C_3_N_4_/WP/W SAs in the dark as well as under simulated sunlight irradiation.


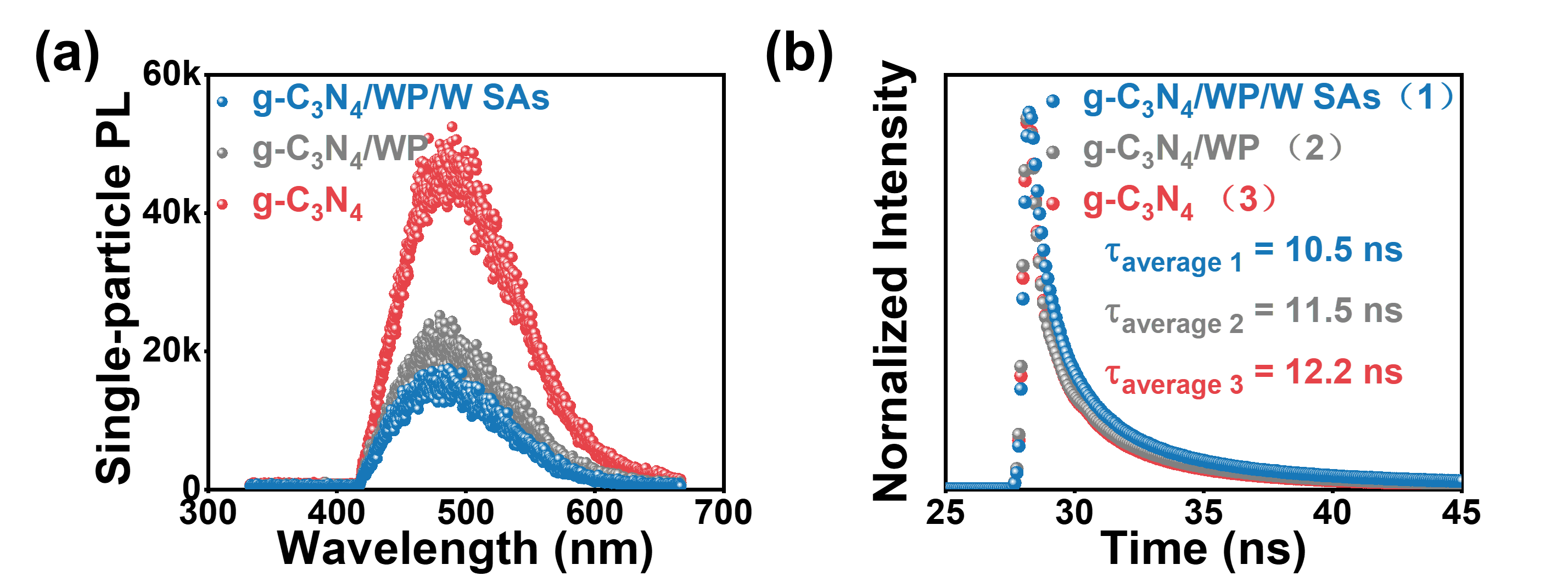


**Figure S19.** (a) Single-particle PL spectra of g-C_3_N_4_, g-C_3_N_4_/WP and g-C_3_N_4_/WP/W SAs. (b) Single-particle PL lifetime maps of g-C_3_N_4_, g-C_3_N_4_/WP and g-C_3_N_4_/WP/W SAs.


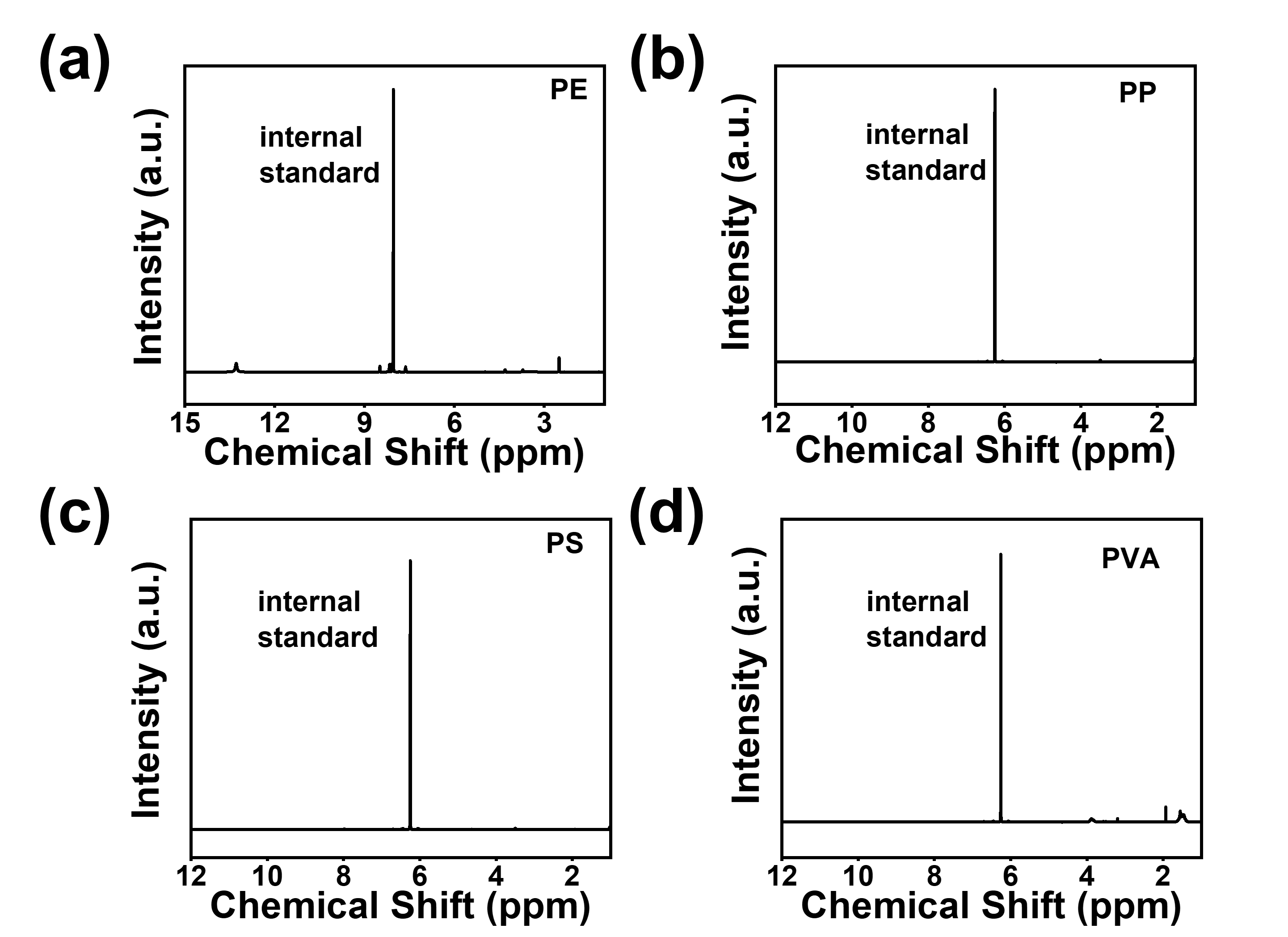


**Figure S20.** ^1^H-NMR spectra of (a) PE, (b) PP, (c) PS and (d) PVA.


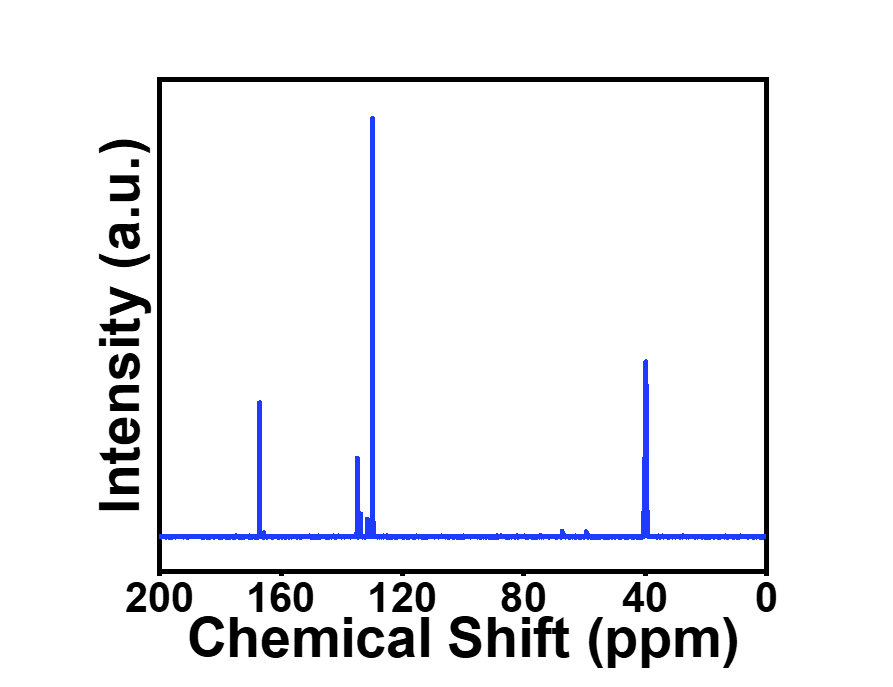


**Figure S21.** ^13^C-NMR spectra of precipitation.


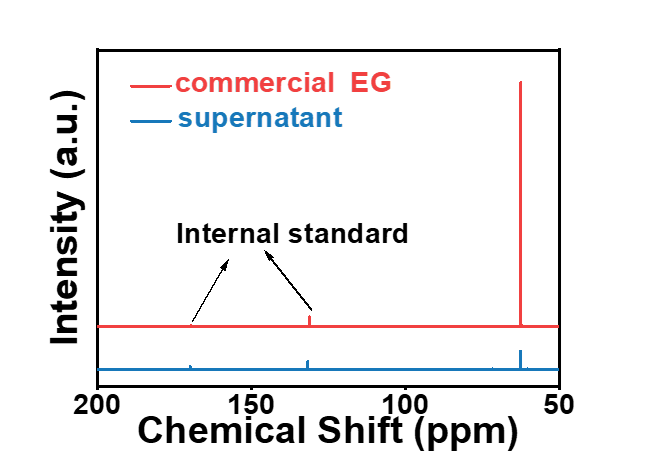


**Figure S22.** ^13^C-NMR spectra of supernatant.


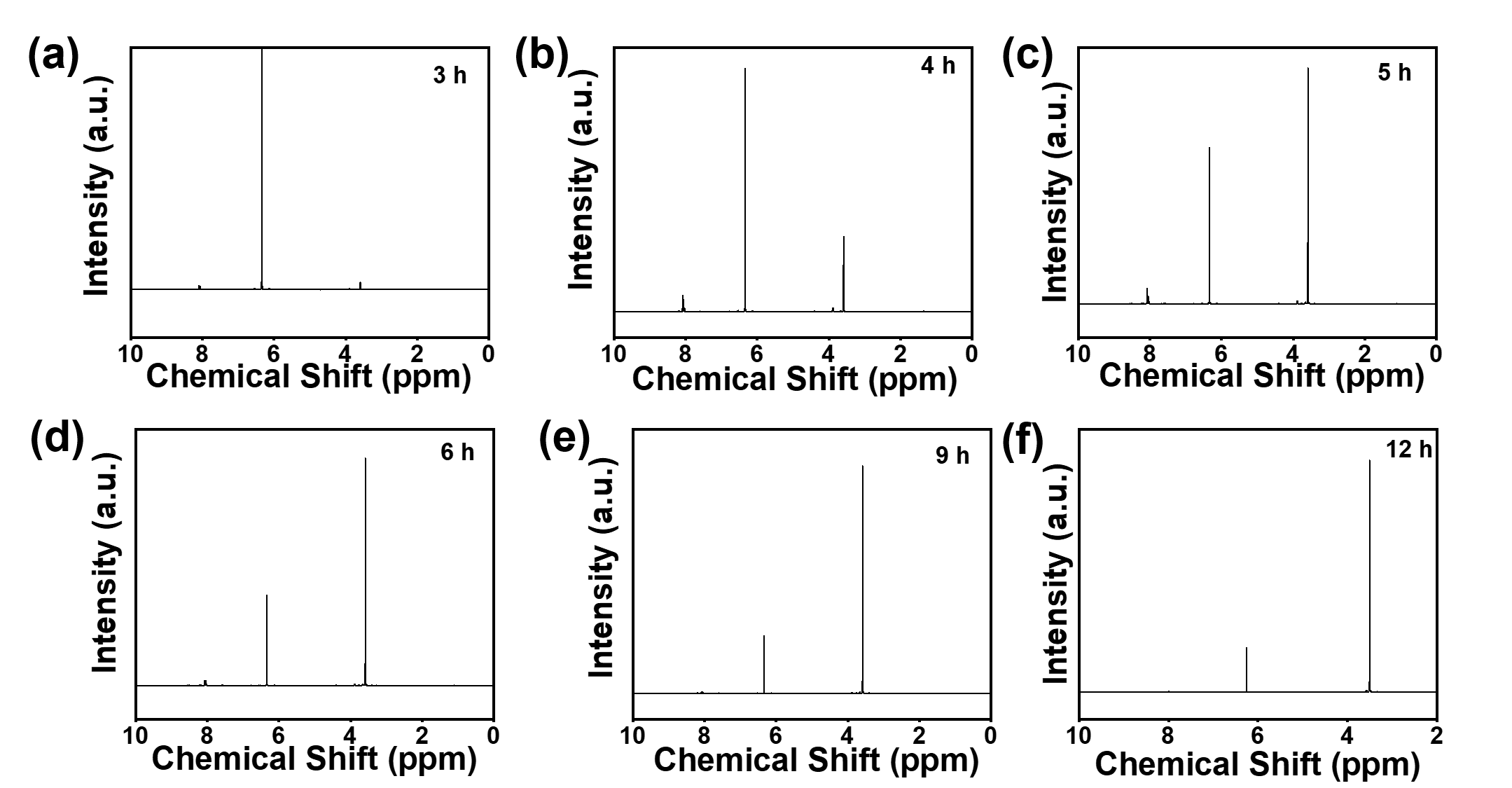


**Figure S23.** ^1^H-NMR spectra of PET for different time (a) 3h, (b) 4h, (c) 5h, (d) 6h, (e) 9h and (f) 12h.


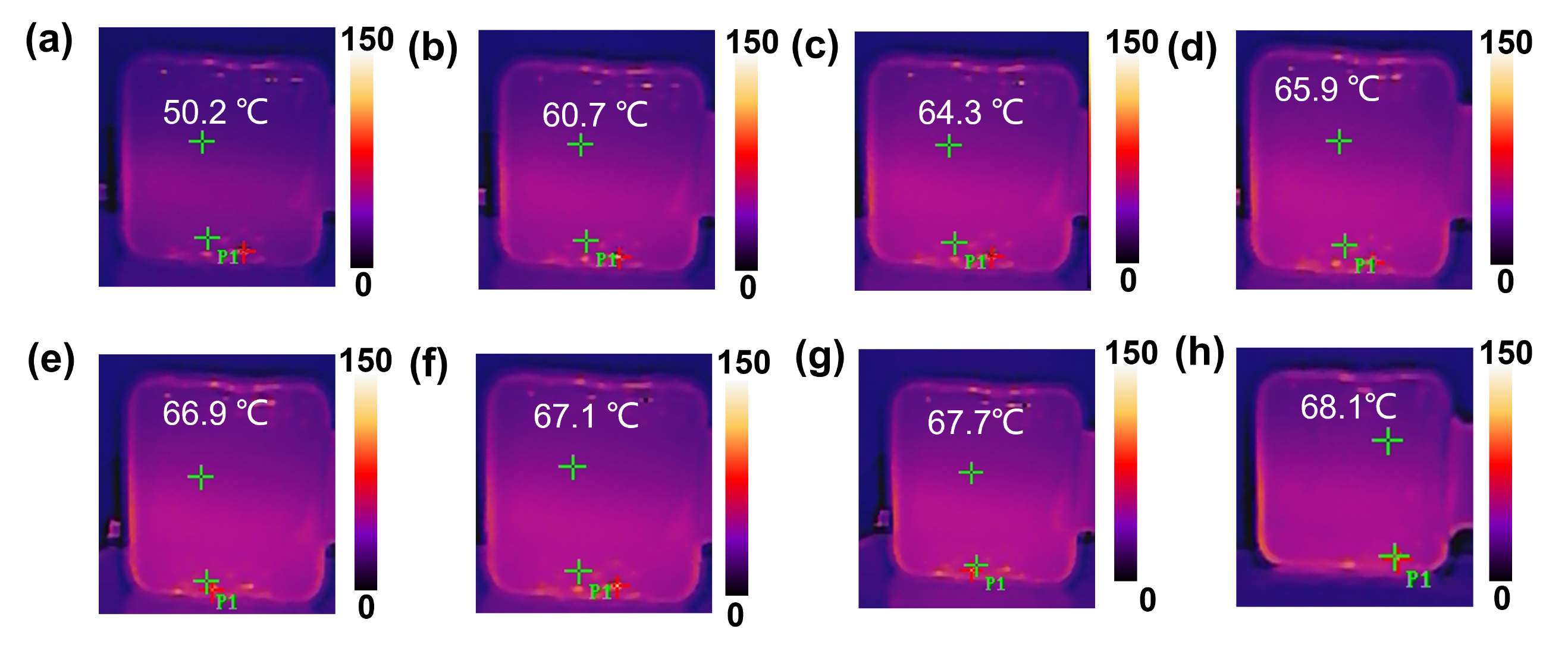


**Figure S24.** IR thermal images of g-C_3_N_4_/WP-3/W SAs at (a) 5, (b) 10, (c) 15, (d) 20, (e) 25, (f) 30, (g) 35 and (h) 40 min.


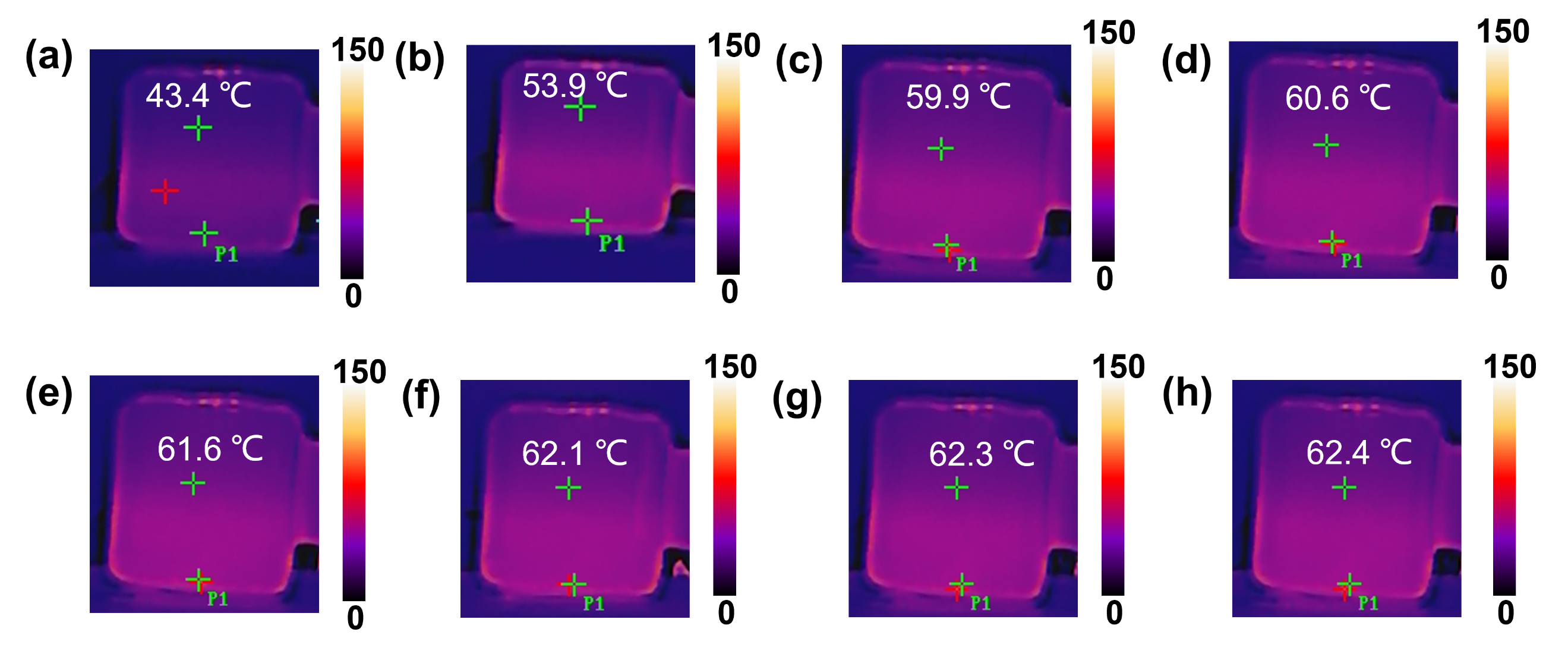


**Figure S25.** IR thermal images of g-C_3_N_4_/WP at (a) 5, (b) 10, (c) 15, (d) 20, (e) 25, (f) 30, (g) 35 and (h) 40 min.


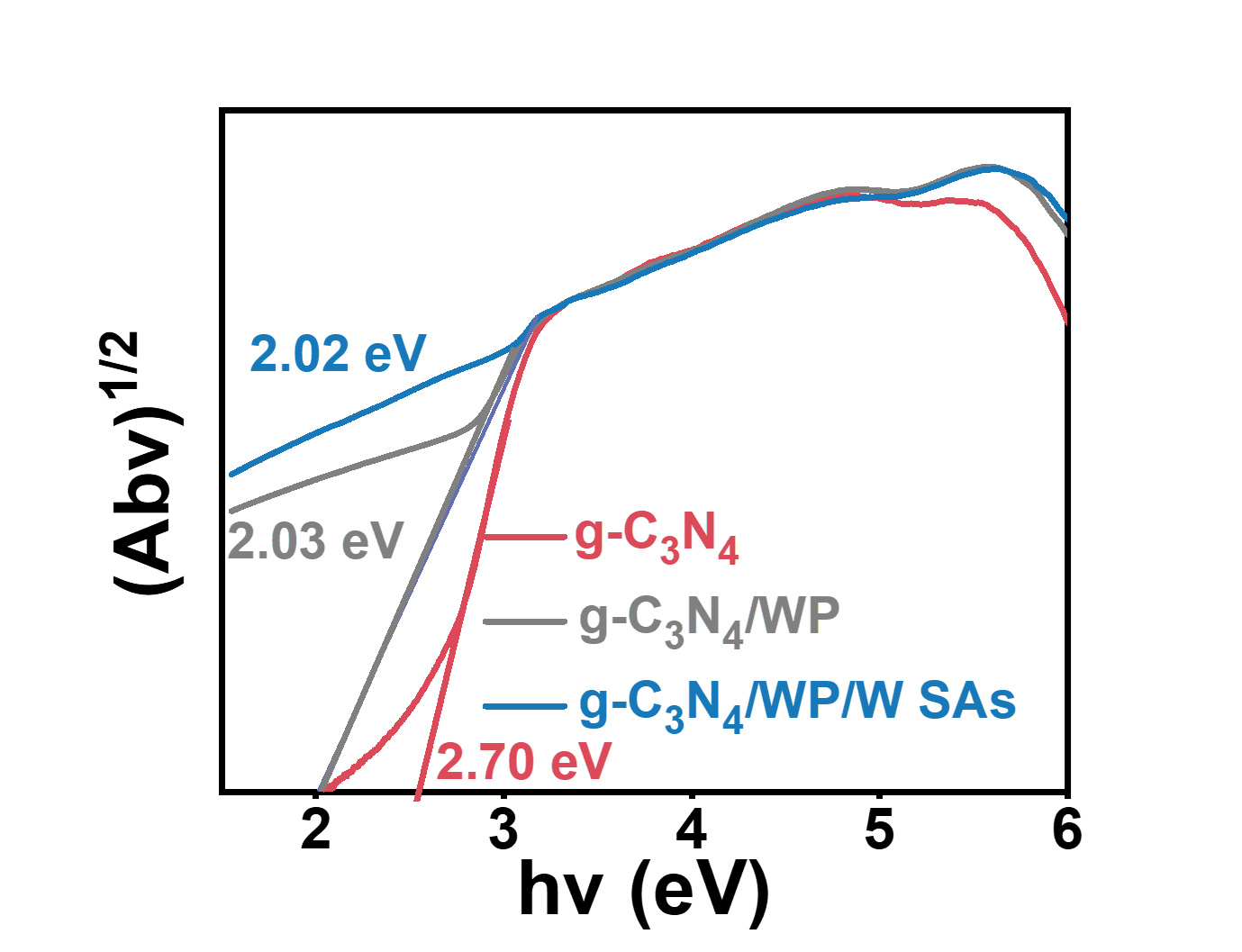


**Figure S26.** The tauc plot for g-C_3_N_4_, g-C_3_N_4_/WP/W SAs and g-C_3_N_4_/WP.


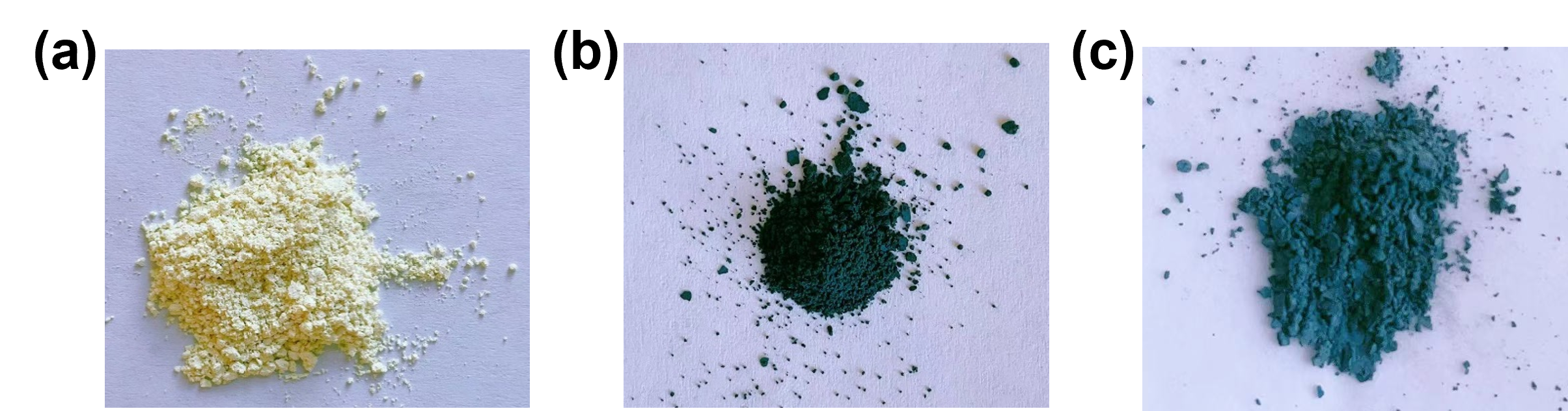


**Figure S27.** The color of (a) g-C_3_N_4_, (b) g-C_3_N_4_/WP/W SAs and (c) g-C_3_N_4_/WP.


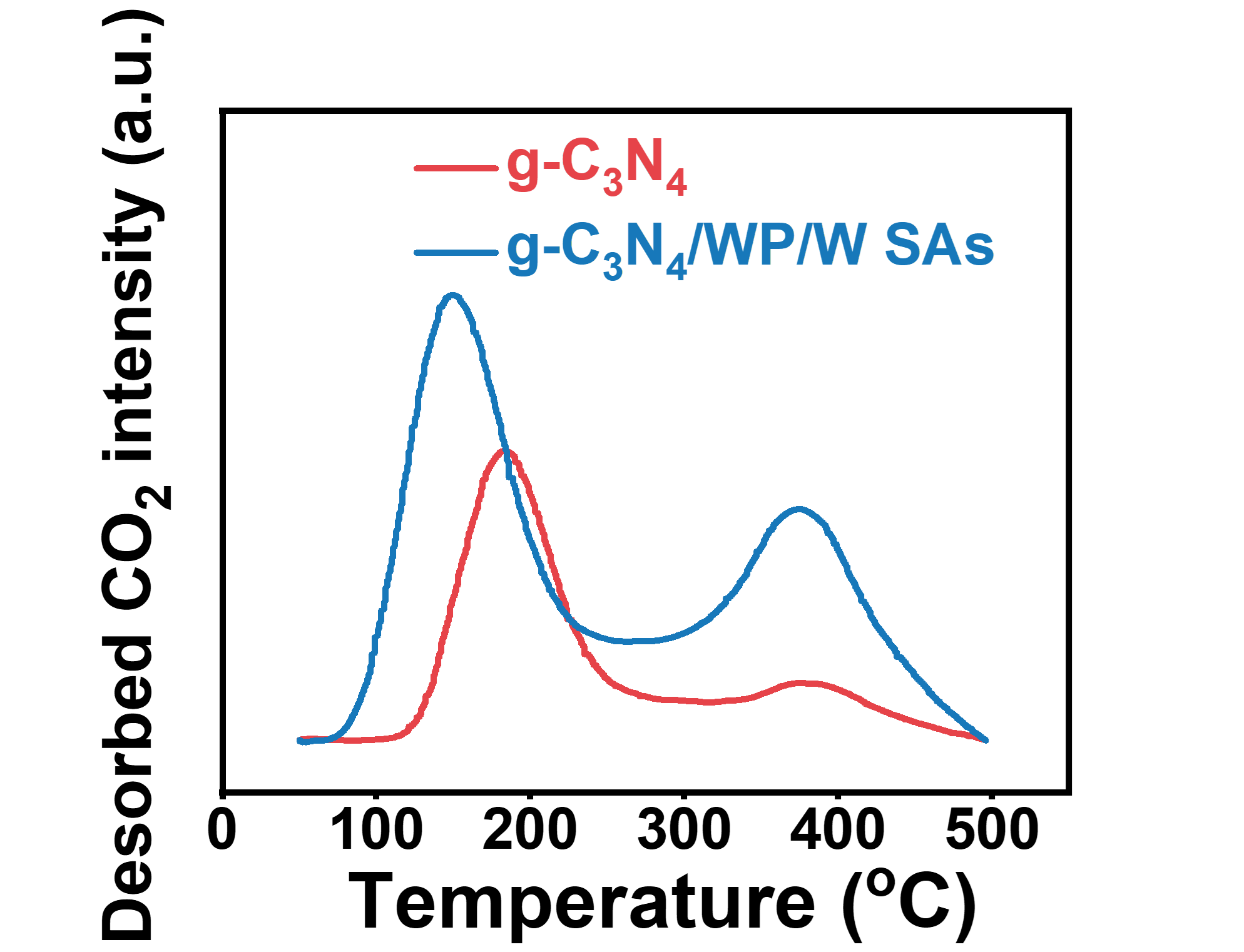


**Figure S28.** The CO_2_-TPD of g-C_3_N_4_ and g-C_3_N_4_/WP/W SAs.


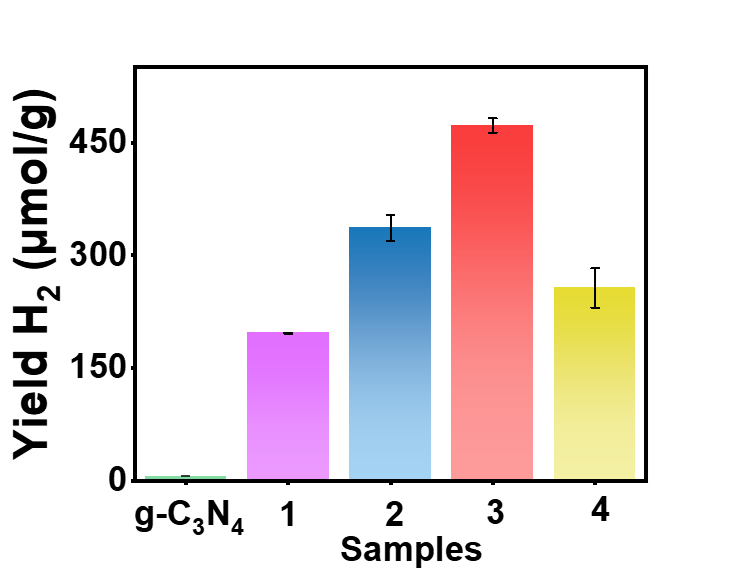


**Figure S29.** Photothermal reforming PET using different samples without temperature control. 1, 2, 3 and 4 represent g-C_3_N_4_/WP-1/W SAs, g-C_3_N_4_/WP-2/W SAs, g-C_3_N_4_/WP/W SAs and g-C_3_N_4_/WP-4/W SAs, respectively.


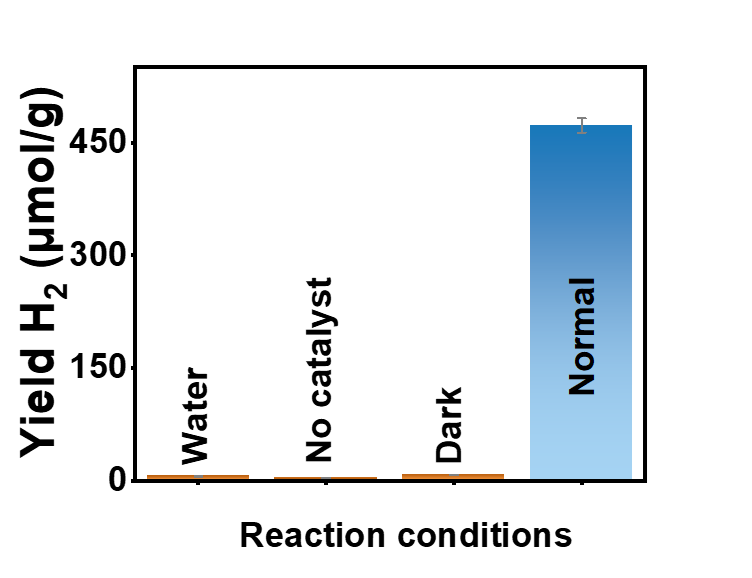


**Figure S30.** The photothermal reforming of PET to H_2_ over g-C_3_N_4_/WP/W SAs under different conditions.


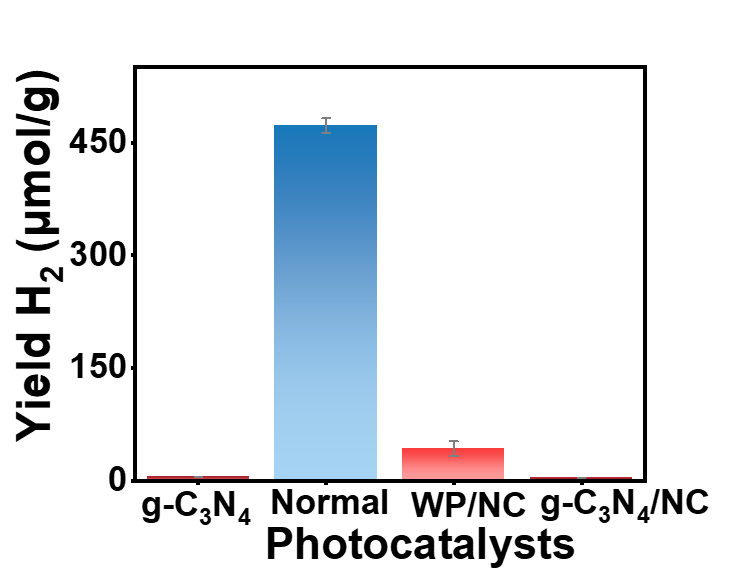


**Figure S31.** Photothermal reforming of PET to H_2_ over different photocatalysts.


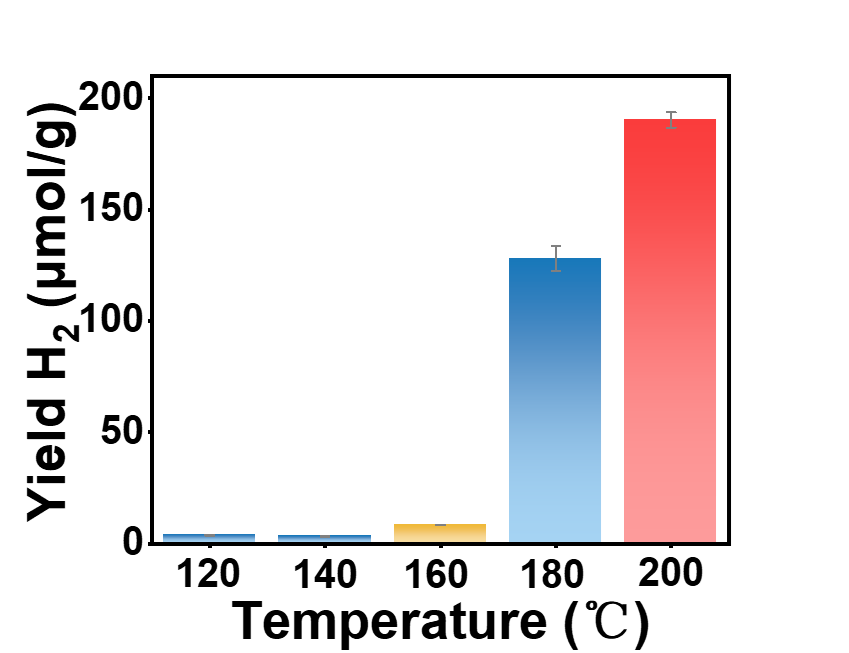


**Figure S32.** The effect of temperature on hydrothermal pretreatment of PET for the photothermal reforming of PET to H_2_ over g-C_3_N_4_/WP/W SAs.


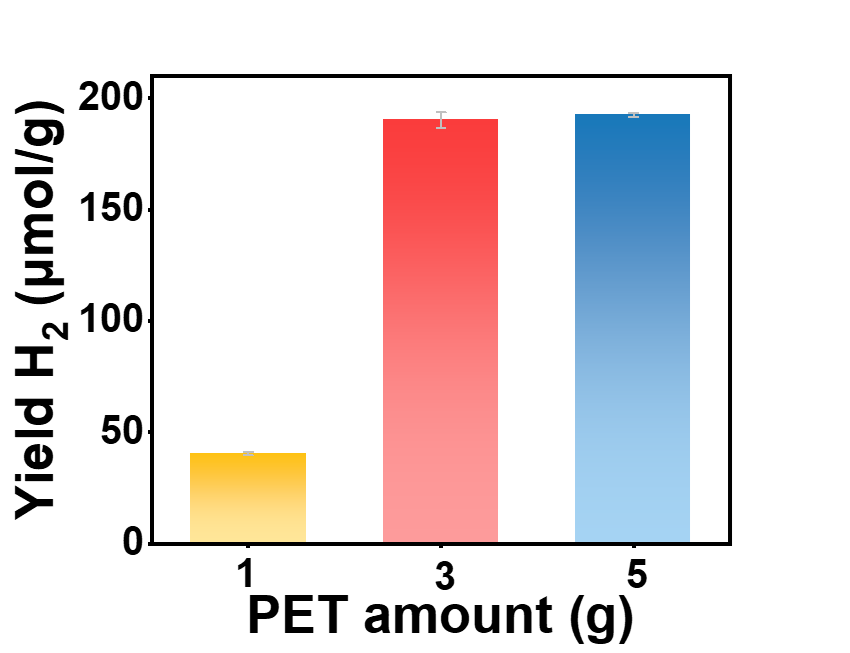


**Figure S33.** The effect of PET amount on hydrothermal pretreatment for the photothermal reforming of PET to H_2_ over g-C_3_N_4_/WP/W SAs.


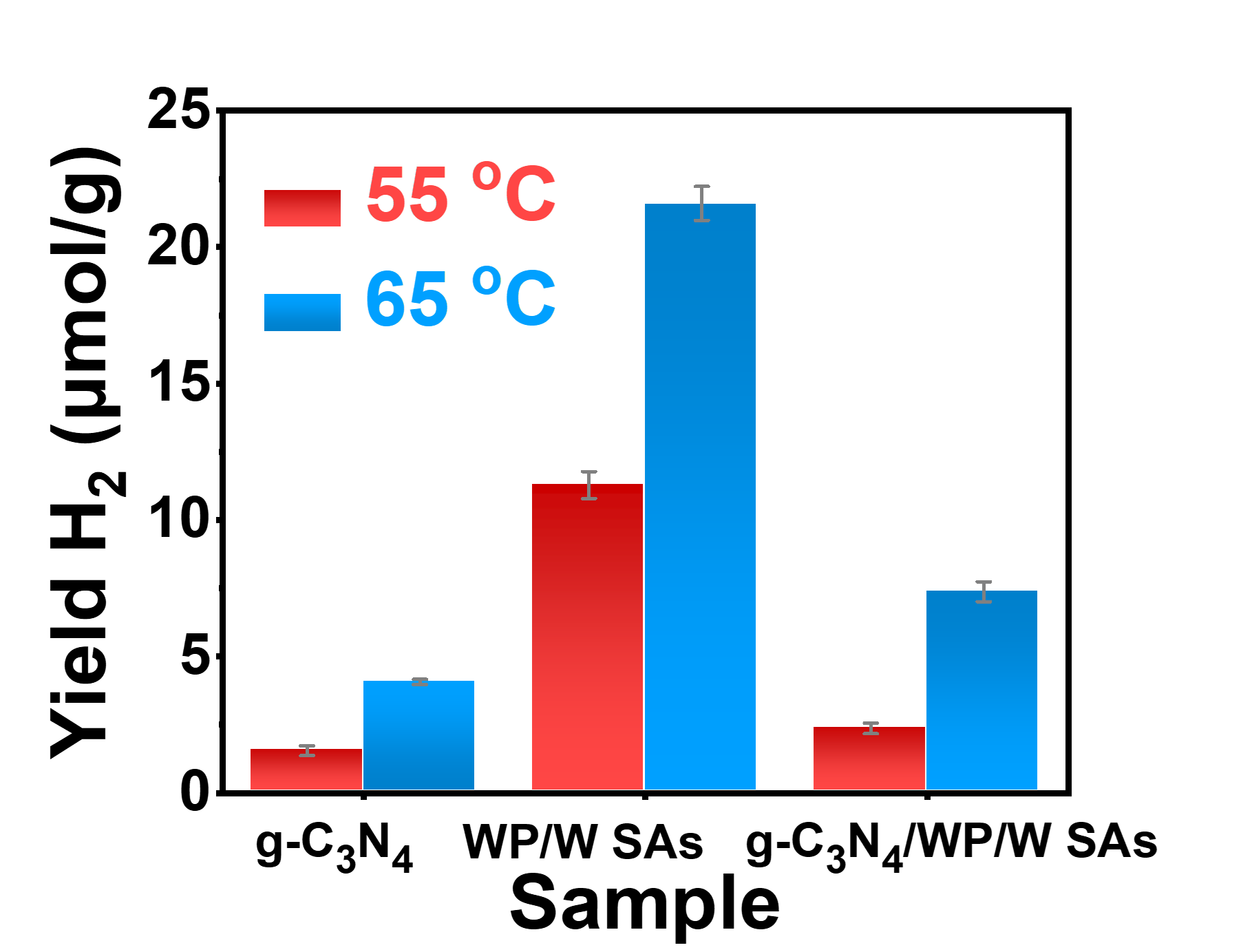


**Figure S34.** Thermal catalytic hydrogen evolution rate of g-C_3_N_4_/WP/W SAs, g-C_3_N_4_ and WP/W SAs at different temperatures.


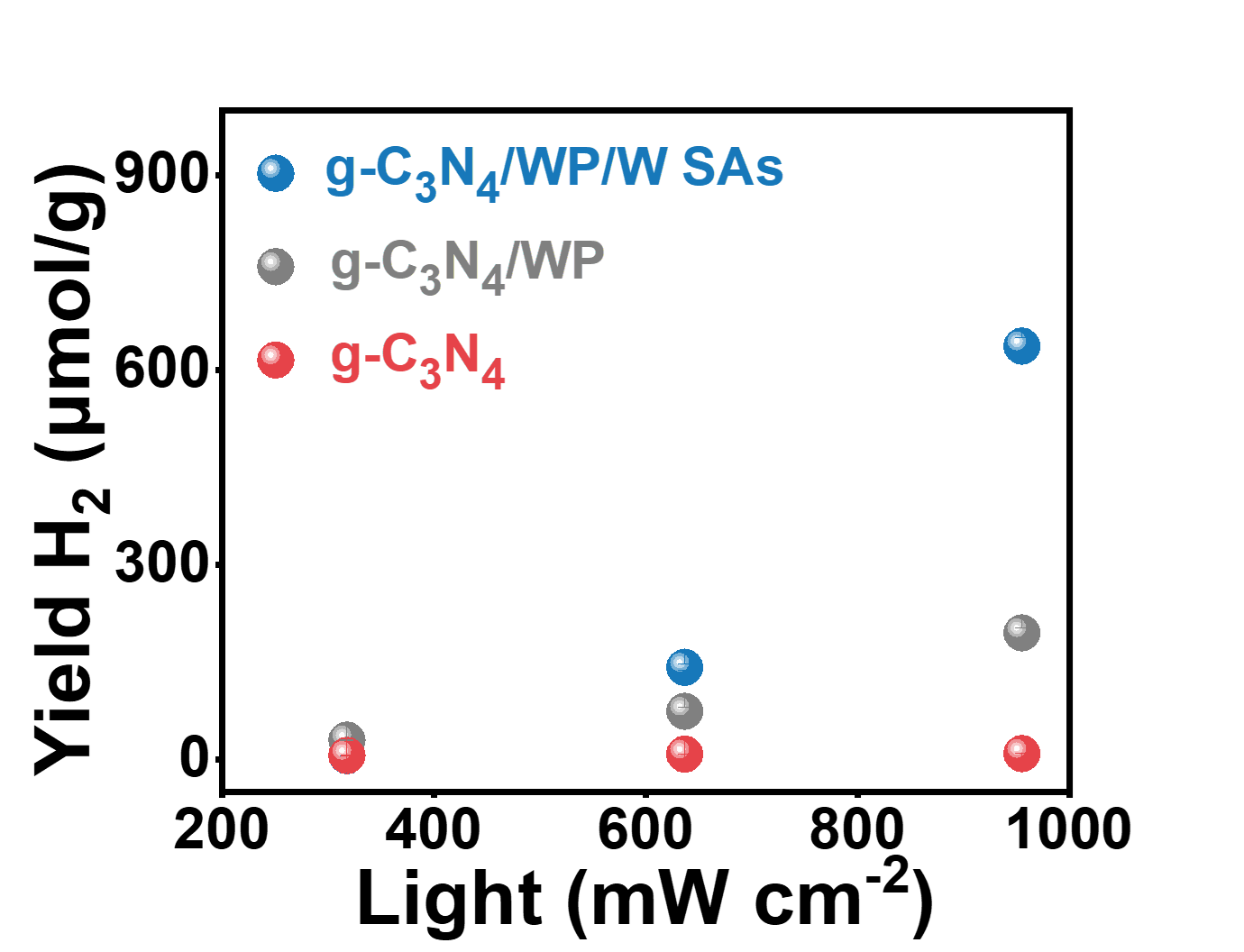


**Figure S35.** Linear relationship between photothermal reforming of PET and light intensity in g-C_3_N_4_, g-C_3_N_4_/WP and g-C_3_N_4_/WP/W SAs.


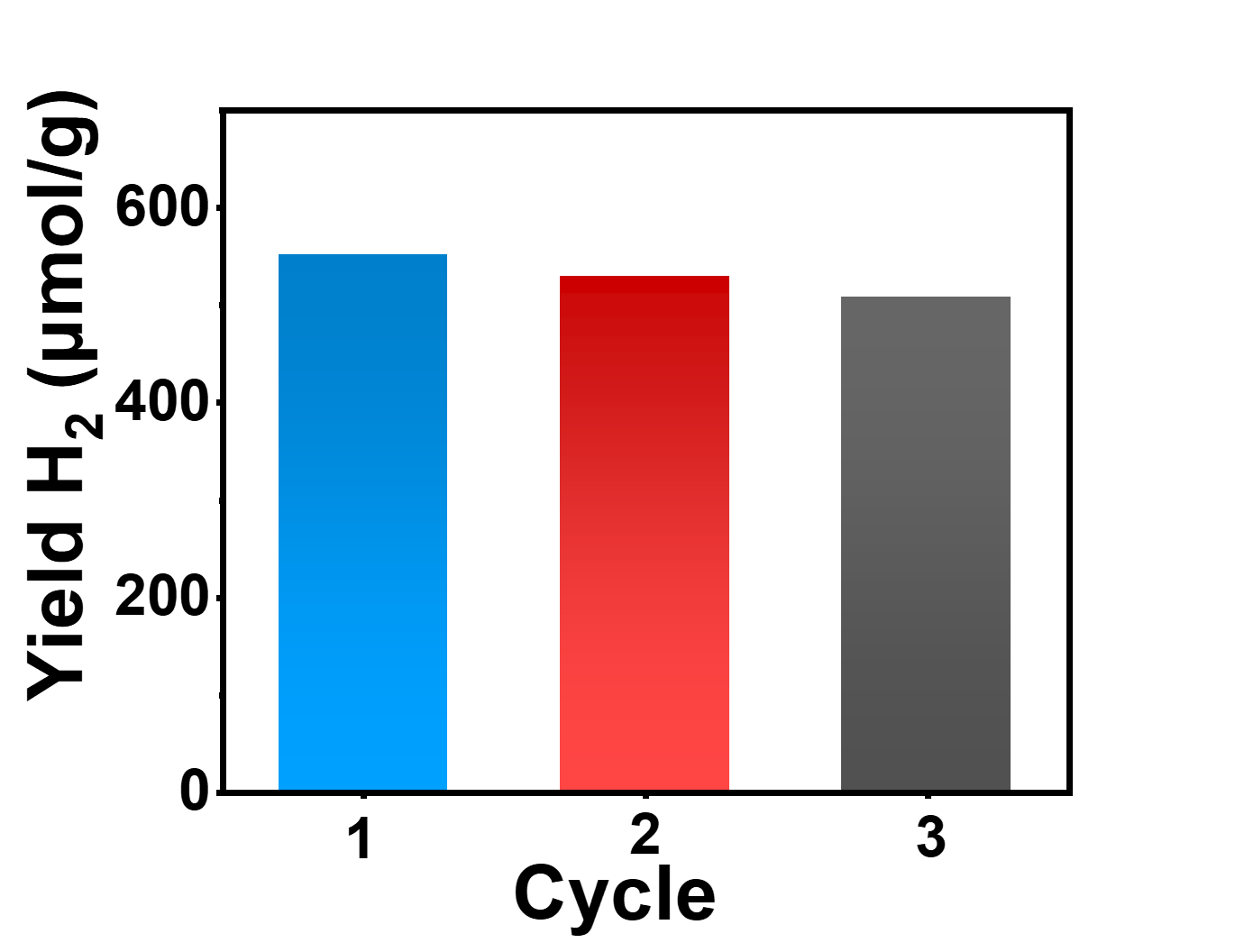


**Figure S36.** Photothermal reforming of PET to H_2_ stability experiment of g-C_3_N_4_/WP/W SAs.


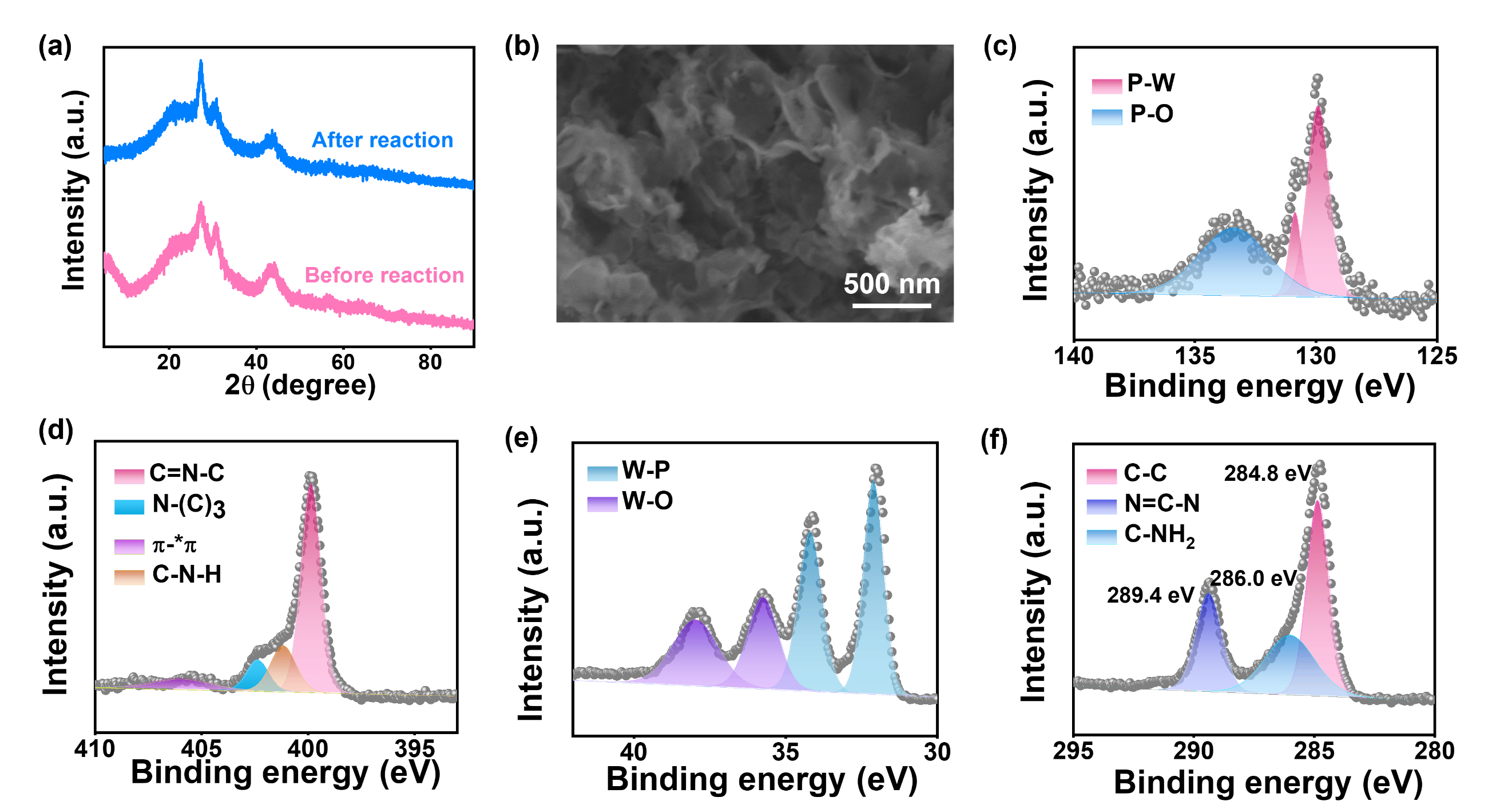


**Figure S37.** (a) XRD pattern of g-C_3_N_4_/WP/W SAs before and after reaction. (b) SEM image of g-C_3_N_4_/WP/W SAs after reaction. High resolution XPS spectra in the region of (c) P 2p, (d) N 1s, (e) W 4f and (f) C 1s of g-C_3_N_4_/WP/W SAs after reaction.


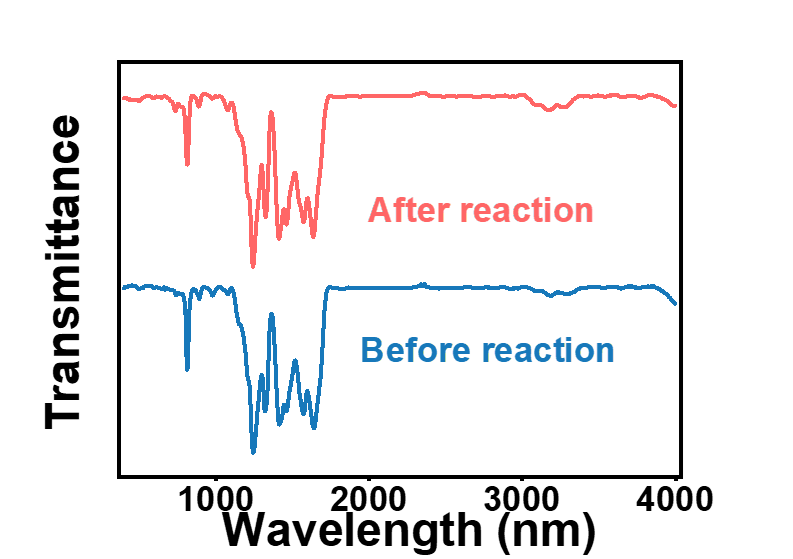


**Figure S38.** FTIR of g-C_3_N_4_/WP/W SAs before and after reaction.

**
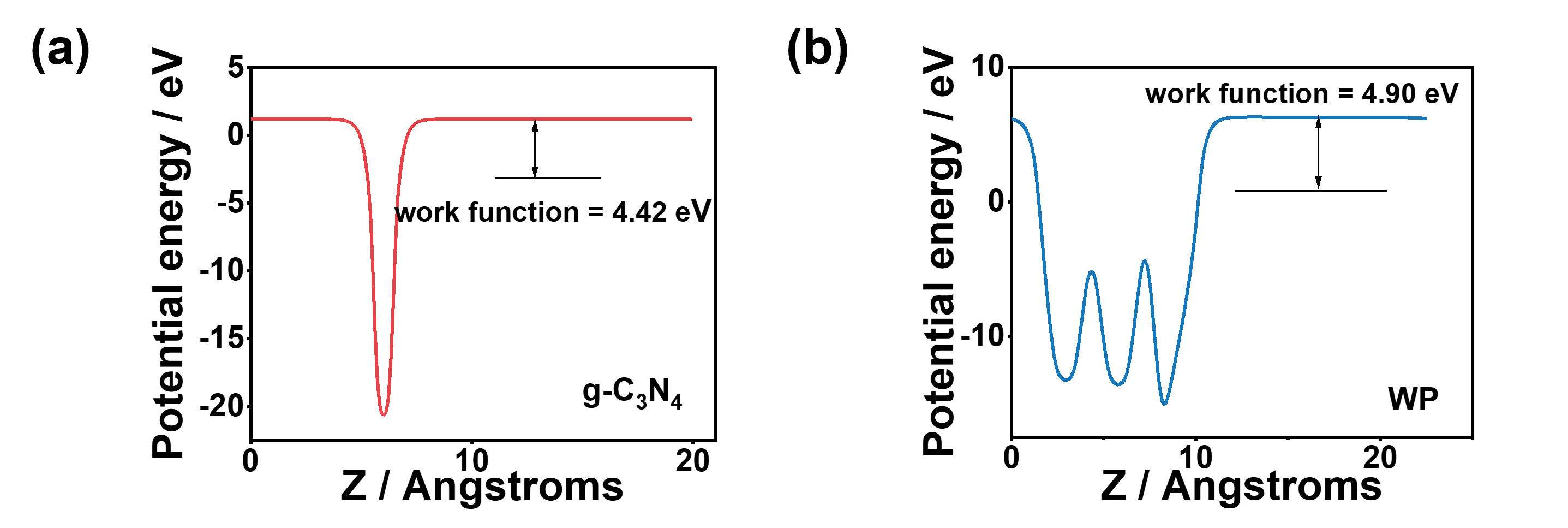
**

**Figure S39.** The work function for (a) g-C_3_N_4_ and (b) WP.


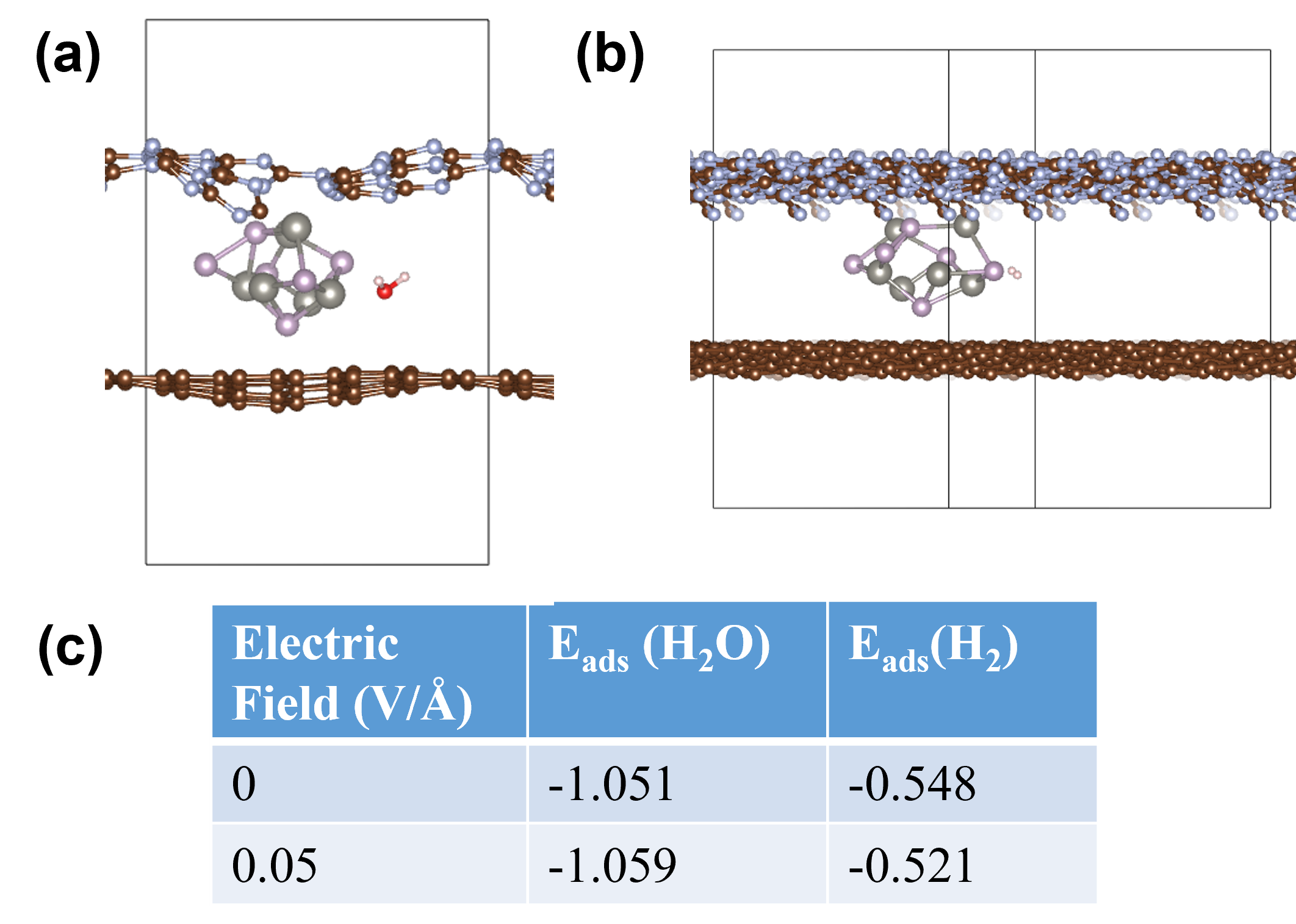


**Figure S40.** (a) Optimized structures of H_2_O adsorbed over g-C_3_N_4_/WP with an electric field of 0.05 V/Å. (b) Optimized structures of H_2_ adsorbed over g-C_3_N_4_/WP with an electric field of 0.05 V/Å. (c) Adsorption energy on g-C_3_N_4_/WP in different conditions.


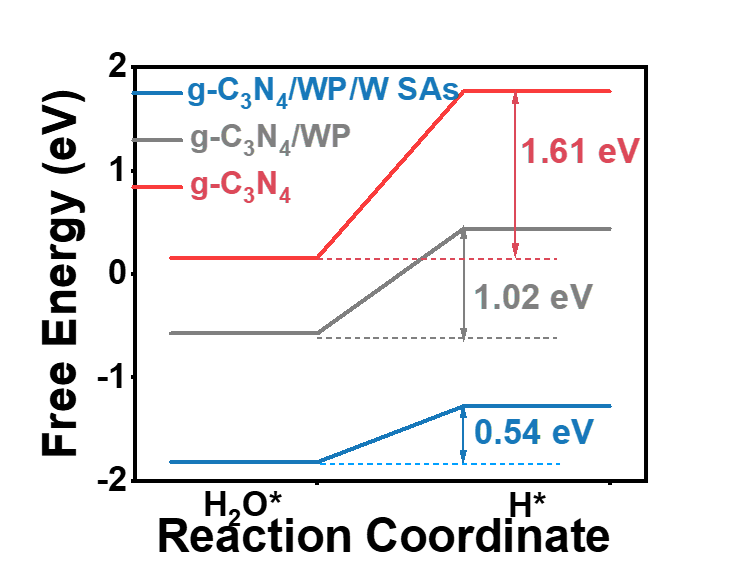


**Figure S41.** Free energy diagram of g-C_3_N_4_, g-C_3_N_4_/WP and g-C_3_N_4_/WP/W SAs.


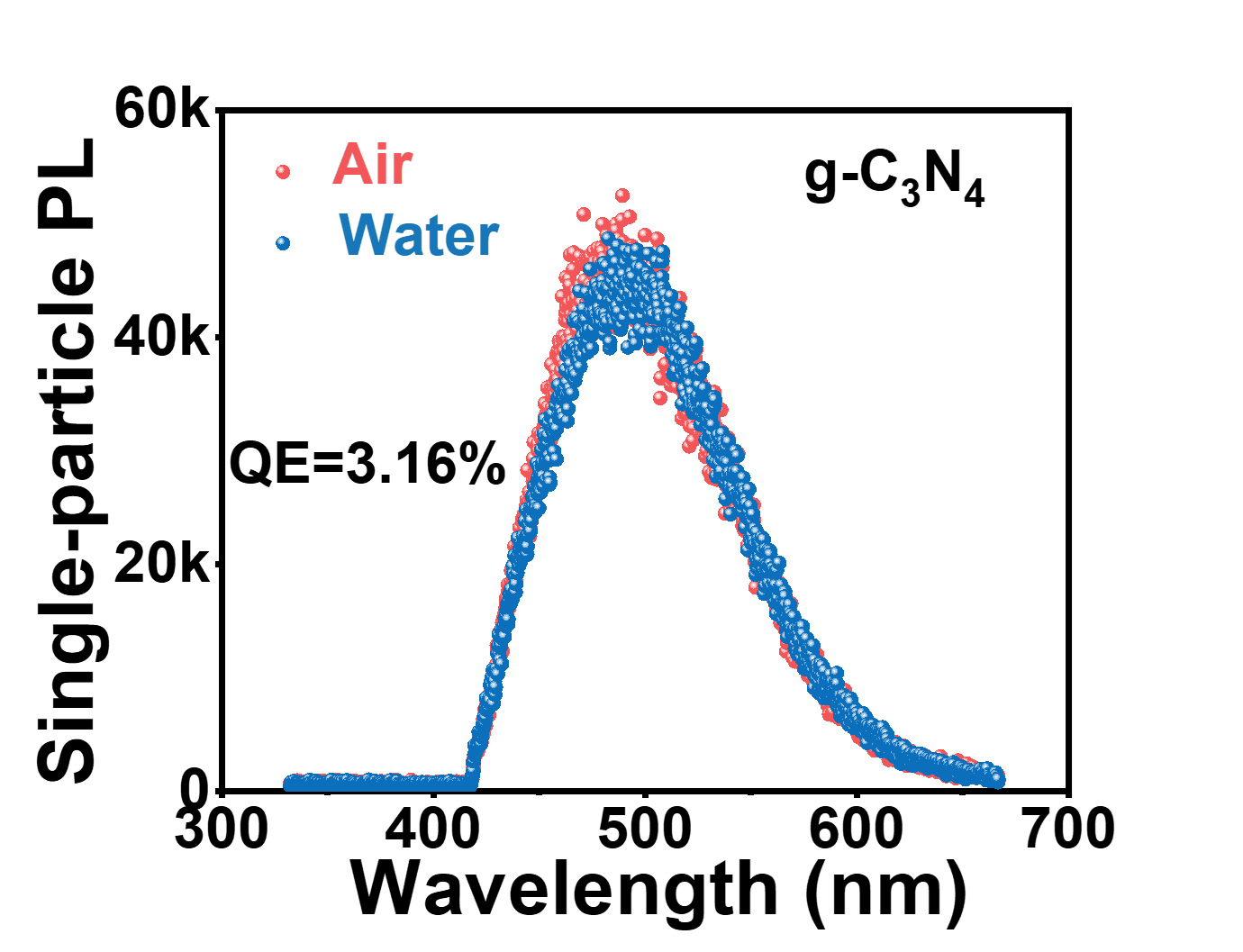


**Figure S42.** Single-particle PL spectra of g-C_3_N_4_.


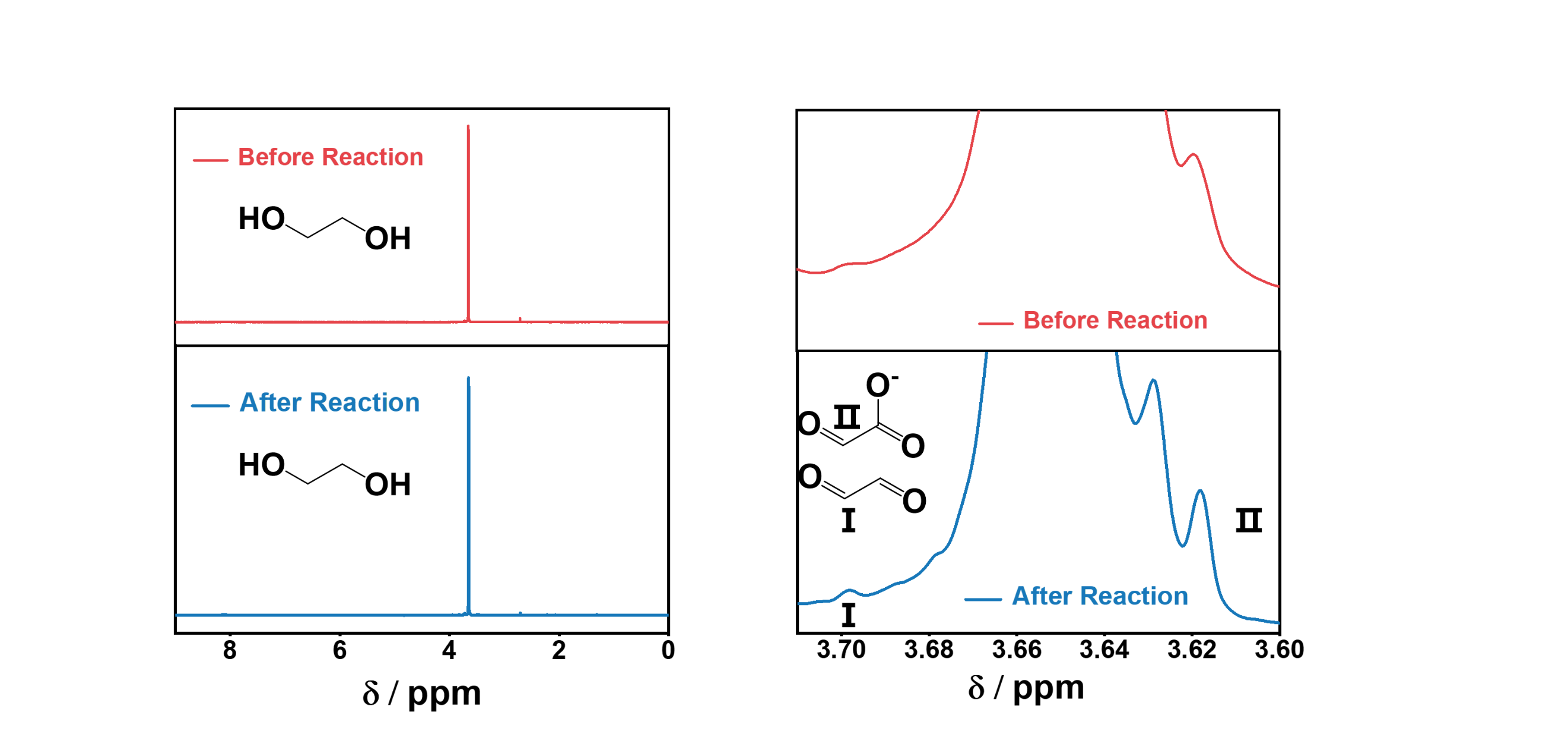


**Figure S43.** ^1^H-NMR spectra of PET before and after 10 h reaction.

**Table S1.** Fitting results of W LIII-edge EXAFS curves.

**
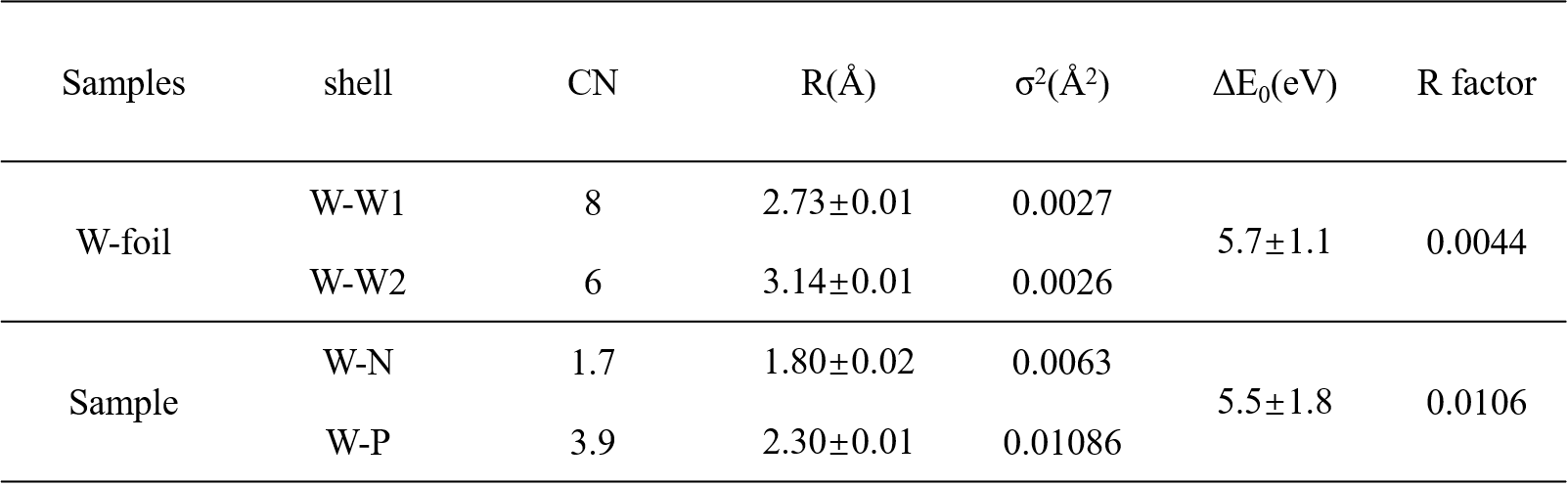
**

**Table S2.** Surface area, pore volume and average pore diameter of g-C_3_N_4_, g-C_3_N_4_/WP and g-C_3_N_4_/WP/W SAs, respectively.


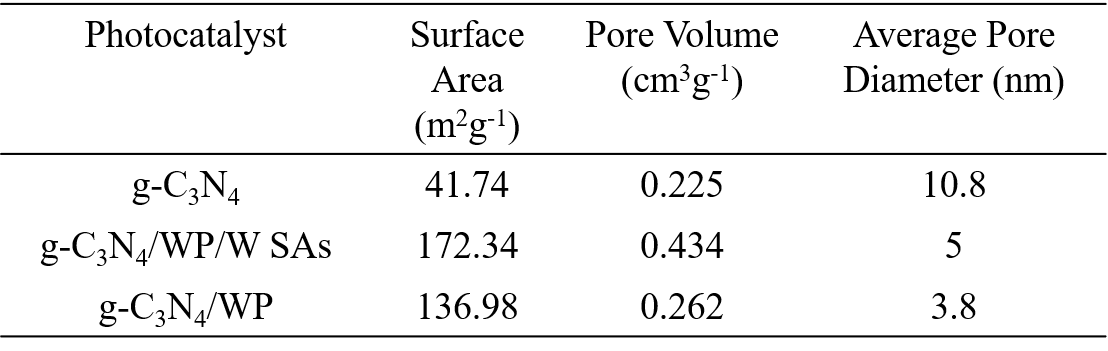


**Reference**

[1] X. Gong, Y. Zhang, Y. Xu, G. Zhai, X. Liu, X. Bao, Z. Wang, Y. Liu, P. Wang, H. Cheng, Y. Fan, Y. Dai, Z. Zheng, B. Huang, *ACS Applied Materials & Interfaces.* **2022**, *14*, 51029-51040.

[2] P. Wang, Z. Zhang, N. Song, X. An, J. Liu, J. Feng, B. Xi, S. Xiong, *CCS Chemistry.* **2023**, *5*, 397-411.

[3] X. Gong, F. Ma, Y. Zhang, Y. Li, Z. Wang, Y. Liu, P. Wang, H. Cheng, Y. Dai, Y. Fan, B. Huang, Z. Zheng, *ACS Catalysis.* **2023**, *13*, 12338-12349.

[4] L. Xu, B. Tian, T. Wang, Y. Yu, Y. Wu, J. Cui, Z. Cao, J. Wu, W. Zhang, Q. Zhang, J. Liu, Z. Li, Y. Tian, *Energy Environ. Sci.* **2022**, *15*, 5059-5068.

[5] T. Liu, W. Zhu, N. Wang, K. Zhang, X. Wen, Y. Xing, Y. Li, *Advanced Science.* **2023**, *10*, 2302503.

[6] X. Li, Y. Huang, W. Ho, S. Han, P. Wang, S. Lee, Z. Zhang, *Applied Catalysis B: Environmental.* **2023**, *338*, 123048.

[7] T. T. Dang, T. K. A. Nguyen, K. C. Bhamu, T. Mahvelati-Shamsabadi, V. K. H. Van, E. W. Shin, K.-H. Chung, S. H. Hur, W. M. Choi, S. G. Kang, J. S. Chung, *ACS Catalysis.* **2022**, *12*, 13763-13780.

[8] P. Błoński, J. Tuček, Z. Sofer, V. Mazánek, M. Petr, M. Pumera, M. Otyepka, R. Zbořil, *Journal of the American Chemical Society.* **2017**, *139*, 3171-3180.
